# Patient‐Derived Microphysiological Systems for Precision Medicine

**DOI:** 10.1002/adhm.202303161

**Published:** 2023-12-10

**Authors:** Jihoon Ko, Jiyoung Song, Nakwon Choi, Hong Nam Kim

**Affiliations:** ^1^ Department of BioNano Technology Gachon University Seongnam‐si Gyeonggi‐do 13120 Republic of Korea; ^2^ Brain Science Institute Korea Institute of Science and Technology (KIST) Seoul 02792 Republic of Korea; ^3^ Division of Bio‐Medical Science & Technology KIST School Seoul 02792 Republic of Korea; ^4^ KU‐KIST Graduate School of Converging Science and Technology Korea University Seoul 02841 Republic of Korea; ^5^ School of Mechanical Engineering Yonsei University Seoul 03722 Republic of Korea; ^6^ Yonsei‐KIST Convergence Research Institute Yonsei University Seoul 03722 Republic of Korea

**Keywords:** microphysiological system, patient‐derived, physiologically‐relevant, precision medicine

## Abstract

Patient‐derived microphysiological systems (P‐MPS) have emerged as powerful tools in precision medicine that provide valuable insight into individual patient characteristics. This review discusses the development of P‐MPS as an integration of patient‐derived samples, including patient‐derived cells, organoids, and induced pluripotent stem cells, into well‐defined MPSs. Emphasizing the necessity of P‐MPS development, its significance as a nonclinical assessment approach that bridges the gap between traditional in vitro models and clinical outcomes is highlighted. Additionally, guidance is provided for engineering approaches to develop microfluidic devices and high‐content analysis for P‐MPSs, enabling high biological relevance and high‐throughput experimentation. The practical implications of the P‐MPS are further examined by exploring the clinically relevant outcomes obtained from various types of patient‐derived samples. The construction and analysis of these diverse samples within the P‐MPS have resulted in physiologically relevant data, paving the way for the development of personalized treatment strategies. This study describes the significance of the P‐MPS in precision medicine, as well as its unique capacity to offer valuable insights into individual patient characteristics.

## Graphical abstract

Microphysiological systems now integrate patient‐derived samples, functioning as physiologically relevant avatars for high‐throughput assays. This evolution addresses an unmet clinical need for a patient‐centered nonclinical assessment platform, steering toward more precise and personalized approaches in medical research and drug development.

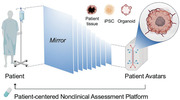

## 1 Introduction

Biomedical research and drug development require accurate and reliable models to study human physiology and disease mechanisms.^[^ ^1^ ^]^ Despite the advantages of traditional models, they often lack the precision needed to represent human organs' complexities. Animal models, valuable for certain aspects, struggle due to interspecies differences,^[^ ^2^ ^]^ while immortalized cell lines lack the physiological relevance of primary cells, limiting their utility in representing human diseases.^[^ ^3^ ^]^ These limitations hinder the translation of preclinical findings into effective clinical treatments.^[^ ^4^ ^]^ To address these limitations, a patient‐derived microphysiological system (P‐MPS) has emerged as a promising alternative. Recent breakthroughs in microfluidic‐based three‐dimensional (3D) cell culture highlight the potential of patient‐specific in vitro test beds. The MPS recreates the microenvironment around specific tissues or organs, incorporating factors like cell–cell interactions,^[^ ^5^ ^]^ extracellular matrix (ECM) composition,^[^ ^6^ ^]^ and fluid flow dynamics.^[^ ^7^ ^]^ Faithful reproduction enables the study of complex disease processes and the assessment of drug responses in a more physiologically relevant context. While some studies have utilized immortalized cell lines in MPS, a notable gap remains between MPS‐generated data and clinical outcomes.^[^ ^8^, ^9^, ^10^ ^]^

**Figure 1:**
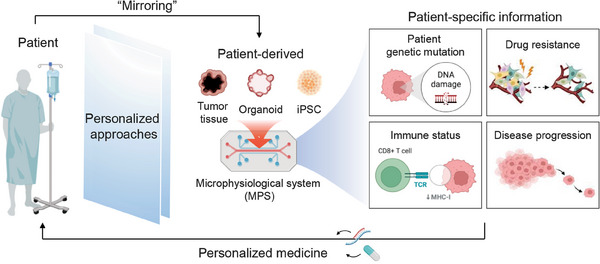
P‐MPS for precision medicine. The illustration describes the application of P‐MPS in precision medicine. P‐MPS enables the mirroring of patient‐specific characteristics by utilizing patient‐derived samples including iPSCs, organoids, and tumor tissues. This platform plays a critical role in accelerating precision medicine by providing valuable insights into an individual such as genetic variants, drug resistance, immune status, and disease progression. The illustration was created using BioRender.

Bridging this gap is a central rationale for the necessity of P‐MPS. There is a growing recognition that variations in genetic makeup, cellular behavior, and disease susceptibility among individuals significantly impact treatment outcomes.^[^ ^11^, ^12^, ^13^ ^]^ P‐MPS effectively addresses these limitations by leveraging patient‐derived cells (PDC), patient‐derived organoids (PDO), and induced pluripotent stem cells (iPSC) to more precisely emulate individual human physiology, organ function, and disease progression.^[^ ^8^, ^9^, ^10^ ^]^ Using human‐derived cells, P‐MPS essentially creates a virtual avatar‐like representation of patient microphysiology (**Figure** **1**). This approach empowers researchers to explore diseases and evaluate potential therapies using cells and tissues derived directly from patients, capturing the inherent variability observed in human populations. In essence, through the integration of PDCs and the development of models that better reflect the human microenvironment, MPS has evolved to provide personalized treatment approaches and facilitate the rapid identification of effective drugs for patients with limited treatment options.^[^ ^14^ ^]^

The FDA Modernization Act 2.0, passed in December 2022, underscores the escalating demand for cutting‐edge in vitro models and the consequent evolution in nonclinical testing approaches.^[^ ^15^ ^]^ This legislation introduced the notion of nonclinical testing, specifically highlighting Organ Chips (OoC) and MPS, while phasing out conventional animal testing in favor of more advanced in vitro models. Essentially, it underscores the pioneering incorporation of contemporary avatar models within a novel framework. This alignment corresponds with the trajectory of P‐MPS and holds the promise of substantially mitigating risks associated with preclinical studies through the adoption of a modern avatar model.^[^ ^16^ ^]^

The concept of MPS is currently being elaborated by many researchers.^[^ ^17^, ^18^, ^19^, ^20^ ^]^ In this review, we scrutinize the evolutionary trajectory of P‐MPS by categorizing the previously uncharacteristically multifaceted MPS into PDCs, iPSCs, and PDOs. Our exploration begins by assessing their technological maturity through microfluidic‐based 3D cell culture platforms, focusing on achievements at the cell line level. Additionally, we pave the way for a thoughtful discussion with readers, shedding light on persistent challenges, such as the scarcity of crucial patient‐derived samples for P‐MPS development and the intricate standardization processes essential for aligning data with clinical and pharmaceutical applications.

## 2 Traditional In Vitro Testing Models

The quest for a model mirroring patient characteristics has a historical background, initially manifesting in the cultivation of cells isolated from patients in a two‐dimensional (2D) environment using dishes. While fundamental, this method primarily serves to comprehend cellular characteristics and reactions to diverse treatments, laying the groundwork for subsequent investigations. However, its limitations become apparent in its incapacity to explore interactions among various cell types.

In a stride toward more precise in vitro models, the introduction of 3D cellular spheroids in the 1950s marked a major leap.^[^ ^21^ ^]^ Originating from co‐culturing chondrogenic and myogenic cells from chick embryos, these spheroids evolved into a modern platform for drug screening. Subsequent endeavors aimed to cultivate PDCs into spheroids, showing a personalized approach to modeling human diseases and therapeutic responses. Spheroids boast simplicity, the ability to host multiple cell types, and resilience to drugs owing to multicellular culture. However, inherent limitations include the absence of a vascular structure, restricted drug penetration, and core necrosis.

Furthermore, patient‐derived xenograft (PDX) models emerged as an alternative technique, involving the direct implantation of patient tumor tissues into immunodeficient mice. This approach proves beneficial for evaluating treatment responses,^[^ ^22^, ^23^, ^24^ ^]^ assessing drug efficacy,^[^ ^25^ ^]^ identifying potential biomarkers,^[^ ^26^ ^]^ and unraveling disease progression and therapeutic targets through molecular profiling and genetic studies.^[^ ^27^, ^28^ ^]^ However, interspecies differences and ethical considerations pose limitations, as mice may not fully replicate the human microenvironment and immune system interactions.^[^ ^29^ ^]^

In recognition of these differences, efforts are intensifying to find more representative cellular models by combining advances in stem cell biology, tissue engineering, and microfluidics. Ultimately, we develop P‐MPS to provide a unique opportunity to scrutinize personalized human biology in a meticulously controlled and physiologically relevant manner.

## 3 Engineering MPS to Combine Relevance and Throughput

The development of a P‐MPS that combines high physiological relevance and throughput is essential prior to the effective utilization of patient‐derived samples.^[^ ^30^ ^]^ By integrating high‐throughput capabilities into P‐MPS, researchers can overcome the existing limitations in screening efficiency and simultaneously screen multiple drug candidates under a multitude of conditions.^[^ ^31^ ^]^ With the development of personalized MPS using advanced cell sources, customizable 3D cell culture systems are urgently needed.^[^ ^32^ ^]^ Furthermore, there is a growing demand for high‐throughput models capable of generating substantial amounts of data within a short timeframe.^[^ ^33^ ^]^ High‐throughput experimental models have emerged as promising solutions for rapid, cost‐effective, and reproducible data generation and analysis.^[^ ^34^, ^35^ ^]^ Achieving high‐throughput processes involves leveraging automation, robotics, and standardized systems and protocols.^[^ ^35^ ^]^ Additionally, the convergence of image processing and analysis with artificial intelligence (AI), which has progressed significantly owing to large‐scale data generation, holds immense potential in this context.^[^ ^36^, ^37^ ^]^

### 3.1 High‐Throughput Microfluidic Cell Culture Platform

The process of fabricating a microfluidic 3D cell culture platform involves photolithography and soft‐lithography, which can be labor‐intensive and time‐consuming.^[^ ^38^ ^]^ Even after device fabrication, it heavily relies on the user conducting the experiments, leading to variations in results among different researchers. Furthermore, as the use of these devices transitions from laboratory‐scale to practical applications in clinical research, there is a growing demand for mass production with high reproducibility. This necessitates the standardization of both the device and its associated processes. Screening numerous compounds in a single device can significantly contribute to reducing time and costs in drug development.^[^ ^39^ ^]^ For a more specific example, consider a patient with cancer. In such cases, expediting the diagnostic process and promptly obtaining results is crucial. Since the lifespan of these patients is often limited, rapid diagnosis and the prompt establishment of appropriate treatment options are essential. Therefore, an efficient high‐throughput screening (HTS) platform is an essential requirement to achieve rapid, consistent, and reliable results.

In response to these challenges, researchers have endeavored to develop techniques for mass production and standardization. One such approach is the use of “Phaseguides,” which are microstructures that guide the flow of liquid in microchannels.^[^ ^40^ ^]^ Phaseguides significantly reduce the variability in microfluidic priming and purging, resulting in more consistent results across a variety of microfluidic devices. This approach allows the precise control of liquid flow within a well‐level area, allowing cell culture‐available hydrogel lanes to be configured along geometrical confinements. Using this technique, 40 individual microvessel cultures were robustly developed in a 384‐well plate.^[^ ^41^ ^]^ Additionally, Phaseguides technology has established, a technology‐specific protocol for the transplantation of liver spheroids and organoids in microfluidic vascular beds with well‐defined specifications.^[^ ^42^ ^]^ They extend the platform to a readily accessible injection‐molded chip. The OrganoPlate culture platform, produced by Mimetas BV in the Netherlands using the phaseguide technique, is commercially available.^[^ ^43^, ^44^ ^]^ This device can be widely used in various applications including 3D cell migration, cell‐to‐cell interaction, and angiogenesis.

Another approach is the open microfluidic capillary systems, which use capillary action to move fluids within an array of open channels without the need for external pumps or power sources.^[^ ^45^ ^]^ Capillary forces play a crucial role in enabling various fluid functions, particularly in the design of open microfluidic devices. These devices have a single wall that serves as a ceiling, enabling unrestricted access to the channel.^[^ ^46^ ^]^ The channel is open along its length, facilitating compatibility with a diverse range of fluids including biological samples. The use of capillary forces in open microfluidics offers versatile and practical solutions for manipulation and analysis.^[^ ^47^ ^]^ Using this technology, the blood–brain barrier (BBB),^[^ ^48^ ^]^ angiogenesis,^[^ ^49^ ^]^ vascularized tumor spheroids,^[^ ^50^, ^51^ ^]^ and immune cell‐mediated cytotoxicity^[^ ^52^ ^]^ were all developed in a 96‐well plate format.

High‐throughput microfluidic platforms aim to reduce variations in results among different researchers while enhancing efficiency for a wide range of applications. For instance, conventional methods for culturing spheroids or organoids, such as the hanging drop technique,^[^ ^53^ ^]^ have been known to produce variable results, highlighting the need for innovative approaches. One notable advancement involves the development of a novel device that simultaneously supports spheroid formation and vascularization by integrating modified hanging drop and open microfluidic systems.^[^ ^54^ ^]^ These efforts hold the potential to minimize sample variations attributed to external factors and offer substantial advantages, particularly in high‐throughput processes. Furthermore, high‐throughput microfluidic systems have been specifically designed for replicating hepatocyte function in vitro.^[^ ^55^ ^]^ This includes the development of a 96 microfluidic array, referred to as the PREDICT‐96 Array, featuring an integrated ultra‐low‐volume recirculating pumping system. The PREDICT‐96 platform adheres to the industry‐standard 96‐well footprint, allowing compatibility with conventional laboratory equipment and standardization.

### 3.2 Highly Compatible, Integrated Experimental Models

**Figure 2:**
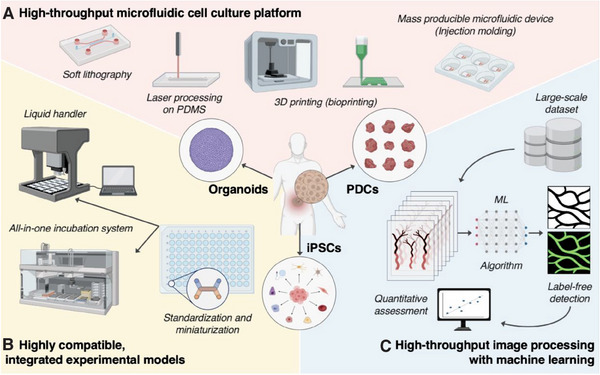
Components for facilitating high‐throughput microfluidic cell culture experiments. A) Diverse efficient fabrication methods for microfluidics devices: soft lithography, laser‐based PDMS direct processing, 3D printing, and injection molding. B) Development of standardized and highly compatible microfluidic devices for integration with automation devices such as liquid handlers and incubation systems. C) Utilize machine learning for analysis of large‐scale data obtained from high‐throughput experiments. Constructing a system for quantitative analysis, including protein expression and morphological features. Streamlining experimental processes through label‐free virtual staining, eliminating the need for fluorescent staining. The illustration was created using BioRender.

Microfluidics‐based 3D cell culture devices differ from traditional labware, like Transwells and well plates, in that they are individually designed by researchers, resulting in diverse formats that make standardizing experimental protocols challenging.^[^ ^56^ ^]^ As a result, these innovative MPS lack standardization in the field, leading to difficulties in the reproducibility, comparability, and translation of microfluidic devices into clinical settings.^[^ ^57^ ^]^ Additionally, these devices have low compatibility with universal tools, such as dispensers and analyzers, owing to differences in specifications. To address this issue, researchers have attempted to devise standardized technologies for mass production (**Figure** **2**A).

To facilitate the utilization of standard equipment and analysis technology, it is advantageous to employ a platform that follows the existing laboratory standard format for easy and efficient analysis. Gerlach et al. developed a plastic microfluidic array comprising 96 capillary electrophoresis channels for HTS and DNA analysis.^[^ ^58^ ^]^ As the array matches the standardized microplate format, it was compatible with existing plate readers and liquid‐handling robotics. This standard plastic microplate format was created through a hot embossing micromachined molding tool. Similarly, an injection‐molded plastic array capable of reproducing the microvasculature within a channel for high‐throughput analysis of vascular phenotypes and drug treatment has been introduced.^[^ ^59^ ^]^ This device conforms to the 384‐well microtiter plate format and allows for obtaining 28 samples per device. As a result, it significantly enhances the compatibility with essential standard laboratory equipment including liquid handling machines, multi‐pipettes, plate readers, and even high‐content imaging machines such as the Yokogawa CQ‐1. This enhanced compatibility streamlines the workflow and ensures seamless integration with existing laboratory processes. Therefore, it is prioritized to make standardization efforts and establish comprehensive guidelines for rapid and large‐scale data processing. Both hot embossing and injection molding have been widely used for mass production of MPS recently, however, those also have limitations due to their high startup costs for mold fabrication, which restricts their practicality for low‐volume production.^[^ ^60^, ^61^, ^62^, ^63^ ^]^ For this reason, they are typically reserved for mass production of finalized designs. Additionally, cutting‐edge 3D printing techniques can be used for prototyping due to their high design flexibility and cost‐effectiveness. This technique offers advantages in creating arbitrary structures, while it has limitations in accurately replicating spatio‐temporal microenvironments.^[^ ^64^, ^65^ ^]^ Regardless of the fabrication methods, standardization of a platform for HTS is an essential requirement to achieve rapid, consistent, and reliable results.

Researchers aim to obtain physiologically relevant results under a wide range of conditions using high‐throughput experimental settings. However, current methods for combining and analyzing multiple organ‐culture systems are time‐consuming, difficult to maintain, and error‐prone, limiting their usefulness in HTS applications. A robotic system‐based compact interrogator has been suggested as an automated tool to facilitate efficient and safe in vitro experiments.^[^ ^35^ ^]^ The system's modular design and programmability enabled seamless execution of experiments on multiple OoCs, ensuring improved efficiency and reliability. The automated culture system facilitated cell imaging on the organ chips, as well as allowed for repeated media transfers, without compromising fluid binding. Additionally, high‐throughput OoC platforms combining programmable fluid flow control and real‐time sensing capabilities can create complex tissue models for drug development workflows.^[^ ^66^ ^]^ These platforms consist of multiple interconnected microfluidic chips, each of which can be independently controlled to create complex tissue models that accurately replicate the in vivo microenvironment. This technology has the potential to accelerate drug development by providing more accurate and efficient testing of new compounds. Finally, a platform integrated with an automated liquid‐handling system enabled high‐throughput organoid culture in microcavity arrays.^[^ ^67^ ^]^ This exemplifies the implementation of automated, high‐content phenotypic screening using large‐scale organoid arrays. Modern progress in high‐throughput experiments and the analysis of in vitro cell culture systems has shown promise in hastening the evolution of medicine and enhancing our understanding of human physiology and disease (Figure 2B).

### 3.3 High‐Throughput Image Processing with Machine Learning

MPS that possess high‐throughput analysis capability plays a critical role in generating large‐scale, high‐quality data necessary for advanced 3D image analysis, enabling the quantitative examination of tissues, cells, and macromolecules. As the number of images increases exponentially with commercialized OoC, manually identifying reads is a labor‐intensive process and is therefore impractical. However, existing software tools, such as ImageJ^[^ ^68^ ^]^ and Cell Profiler,^[^ ^69^ ^]^ were primarily designed for 2D assays, lack proficiency in handling 3D cultured microtissues, and frequently yield inconsistent data analysis results. Therefore, establishing robust assessment parameters and developing state‐of‐the‐art analytical methods are essential for high‐throughput analyses.

Fortunately, advancements in AI have revolutionized data visualization strategies and big data analysis, allowing real‐time and reliable interpretation of diverse data types.^[^ ^70^ ^]^ Machine learning (ML) technologies have emerged across various scientific fields, resulting in significant advancements and innovations. This is particularly evident in the field of image processing, where ML plays a pivotal role.^[^ ^71^ ^]^ With the development of complex algorithms and large datasets, ML algorithms are now capable of analyzing and interpreting images with remarkable accuracy and efficiency.^[^ ^72^ ^]^ In this regard, there is unlimited potential to utilize and develop these advanced algorithms, together with the datasets obtained through MPS experiments.^[^ ^73^ ^]^ Currently, there are ongoing efforts to incorporate machine learning techniques, such as convolutional neural networks (CNN), generative adversarial networks, and other relevant models, into MPS‐based data analysis.

Supervised machine learning algorithms commonly employed in HTS,^[^ ^74^ ^]^ rely on extensive and high‐quality training datasets to extract quantitative image features for compound classification and drug candidate assessment.^[^ ^75^ ^]^ Furthermore, deep learning (DL) models can autonomously extract features from raw inputs using artificial neural networks, making them valuable for analyzing massive amounts of data from individual 3D cultures such as spheroids and organoids.^[^ ^76^ ^]^ For instance, DL models have demonstrated efficacy in classifying spheroid viability after drug exposure and in identifying subtle differences in epithelial spheroid morphology using bright‐field images.^[^ ^77^ ^]^ In vitro multicellular co‐culture arrays were integrated with a support vector machine (SVM) and principal component analysis (PCA) to classify 11 drugs into two categories based on a multi‐metric analysis of cell‐based readouts: those that are skin sensitizing and those that are not. Similarly, antigen‐presenting cell activation was determined by quantifying CD86 and caspase‐3/7 expression in U937 cells. The predictive performance of the SVM classification algorithm was evaluated, achieving a high specificity of 75%, accuracy of 87.5%, and sensitivity of 100%. Those results indicated that incorporating machine learning into image processing and feature extraction is essential for automated, unbiased, and HTS. Additionally, deep learning techniques, particularly CNNs, have played a pivotal role in enhancing the analysis of 3D tumor culture environments.^[^ ^76^, ^78^ ^]^ A high‐throughput microfluidic system for monitoring immune cell infiltration and cytotoxicity within 3D tumor culture environments was introduced, implementing a CNN to extract features based on the pattern of immune cell infiltration for evaluating the efficacy of drugs.^[^ ^79^ ^]^ Both approaches successfully demonstrated the integration of MPS with machine‐learning‐based data analysis. These efforts aim to provide deeper insights and accurate predictions for large volumes of data while reducing analysis time and achieving exceptional accuracy levels.

In addition to these advancements, we introduced a label‐free virtual immunohistochemical (IHC) staining method that leverages DL techniques.^[^ ^80^ ^]^ This innovative approach revolutionizes the traditionally time‐consuming and expensive process of analyzing protein overexpression in cancer tissues using conventional IHC staining. Furthermore, it achieves label‐free cell classification of cancerous cells from white blood cells by combining feature extraction and DL algorithms.^[^ ^81^ ^]^ The study also involved a comparison of the performance of various machine learning algorithms, including deep neural networks, SVMs, logistic regression, and naïve Bayes. The results significantly reduce both the cost and time required for cell staining while greatly enhancing the efficiency and accuracy of cell sorting. Furthermore, this method holds potential for applications in holography, fluorescence lifetime imaging, and Raman microscopy.

The integration of high‐throughput MPS and advanced AI‐based image analysis techniques facilitates the morphological analysis of complex 3D microstructures and streamlines experimental procedures while minimizing errors introduced by human intervention. These analyses will continue to evolve and become more affordable as additional datasets emerge, paving the way for accessible and efficient analytical tools (Figure 2C).

## 4 Balancing Physiological Relevance within MPS

In the earlier section, we investigated crucial engineering considerations aimed at propelling microfluidic‐based 3D cell culture devices toward their evolution into high‐throughput experimental models. Now, we shift our focus to strategies for effectively recapitulating complex biological phenomena within advanced in vitro cell culture devices. So far, researchers have primarily utilized immortalized cell lines or primary human cells to emulate phenotypic features of the in vivo microenvironment in vitro.^[^ ^3^ ^]^

**Figure 3:**
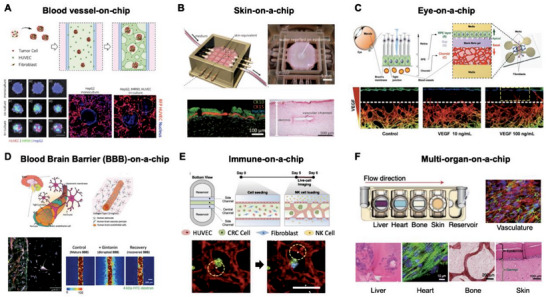
Representative models of MPS for tissue modeling. A) A preferred blood vessel‐on‐a‐chip design that facilitates perfusion through multicellular tumor spheroids to enhance the efficacy of anti‐cancer drug delivery. Red: RFP‐HUVEC, Blue: Nucleus. Reproduced with permission from Park et al. (Lab on a chip 22, 22, (2022); Copyright 2022, Royal Society of Chemistry).^[^ ^88^ ^]^ B) Skin‐on‐a‐chip with vascular integration for investigating vascular absorption. HE‐stained and immune‐stained epidermal layers. Reproduced with permission from Mori et al. (Biomaterials 116 (2017); Copyright 2021, Elsevier).^[^ ^95^ ^]^ C) Blood‐retinal barrier‐on‐a‐chip for simulating pathological angiogenesis and assessing the effectiveness of treatments for choroidal neovascularization. Images show VEGF treatment with and without bevacizumab. Bevacizumab cotreatment inhibits angiogenic sprouting. Red: HUVEC (Anti‐human CD31 Alexa Fluor 647 conjugated). Reproduced with permission from Chung et al. (Advanced Healthcare Material 7, 2, (2018); Copyright 2018, Wiley).^[^ ^92^ ^]^ D) Blood–brain barrier (BBB)‐on‐a‐chip platform for assessing the delivery of BBB‐opening agents through the microvasculature. Reproduced with permission from Seo et al. (Advanced Functional Materials 32(10) (2022); Copyright 2022, Wiley).^[^ ^99^ ^]^ E) Real‐time monitoring of natural killer (NK) cell infiltration and cytotoxicity in a tumor vasculature‐on‐a‐chip. Red: HUVEC (lectin), Green: Colorectal cancer cell (EpCAM), White: NK cells (CellTrace Far Red), Blue: Dead cell (Cytox Blue). Reproduced with permission from Song et al. (Frontiers in Immunology 12,1 (2021); Copyright 2021 Frontiers).^[^ ^101^ ^]^ F) Multi‐organ‐on‐a‐chip with tissue‐specific niche enabling cross‐talk between organs within the system through the vascular circulation (upper), and immunofluorescence or immunohistochemical images for liver, heart, bone, skin, and vasculature (lower). Reproduced with permission from Ronaldson‐Bouchard et al. (Nature Biomedical Engineering 64,4 (2022); Copyright 2022, Springer Nature).^[^ ^105^ ^]^

Many in vitro models, including organoids, aspire to create a morphologically and functionally sophisticated 3D vascular network, a crucial component responsible for the transport of oxygen, nutrients, and cells among organs through intricately lumenized channels.^[^ ^82^, ^83^, ^84^ ^]^ MPS stands out for its unique capacity to mimic a vascular‐like structure with relative simplicity—just by culturing endothelial cells alongside other cell types in a predefined microfluidic area.^[^ ^85^, ^86^ ^]^ The co‐culture of endothelial cells and fibroblasts resulted in the self‐assembly of the 3D microvascular network, giving rise to the human blood vessel‐on‐a‐chip system (**Figure** **3**A).^[^ ^87^, ^88^ ^]^ This microvascular network allows for the assessment of mass transfer and molecule permeability through defined inlet and outlet parameters. Moreover, it facilitates the modeling of angiogenesis mechanisms, essential in the pathological growth of blood vessels, within disease models such as tumor microenvironments (TME),^[^ ^89^, ^90^ ^]^ ocular vascular networks,^[^ ^91^, ^92^ ^]^ and bone microenvironments.^[^ ^93^, ^94^ ^]^

In the field of in vitro skin modeling, traditionally represented primarily by epidermal cells, has achieved a remarkable layered structure, even simulating a subcutaneous vascular network through MPS.^[^ ^95^, ^96^, ^97^ ^]^ This “Skin‐on‐a‐chip” faithfully reproduces the dermis, epidermis, and subcutaneous regions, complete with a perfusable vascular network akin to human skin (Figure 3B).^[^ ^95^ ^]^ The vascularized skin model offers a precise and quantitative assessment of transdermal absorption and transport properties, a benefit for drug absorption and toxicity assessment.

Expanding the scope of MPS to organs with intricate structures, the “eye‐on‐a‐chip” tackles the challenge of replicating the convex hemispherical shape of an eyeball. Utilizing a contact‐lens‐shaped porous scaffold crafted through 3D printing and incorporating a mechanically operated elastic eyelid, this design enables the open‐and‐shut motion of the human eye. Beyond its mechanical creative, the “eye‐on‐a‐chip” has demonstrated potential for pathophysiological modeling including studying conditions like dry eye syndrome.^[^ ^98^ ^]^ Barrier models within this approach capture the layered structure with precision, exemplified by the successful in vitro mimicry of choroidal neovascularization in the retinal pigment epithelium (RPE)‐choroid complex (Figure 3C).^[^ ^92^ ^]^ The use of hydrogels allows the development of a 3D ocular barrier model, replicating Bruch's membrane and inducing angiogenesis in choroidal vessels. This model has been instrumental in assessing the antiangiogenic effects of drugs, like bevacizumab, shedding light on its impact not only during ongoing angiogenic sprouting but also after penetration of the RPE by angiogenic sprouts. The BBB, a crucial barrier function, serves as a highly selective transport barrier necessitating precise assessment of its function. The BBB governs the transport of essential substances while impeding harmful substances' entry. The challenging delivery of medications targeting brain diseases due to the BBB's presence prompted the creation of an in vitro glioblastoma model incorporating the BBB to validate the effects of BBB‐opening agents (Figure 3D).^[^ ^99^ ^]^ This model revealed that BBB‐composing cells induce glioblastoma cell invasion into the surrounding matrix, conferring chemoresistance to drug treatment. Another study utilized the in vitro BBB model to investigate nanoparticle transport in vascular and perivascular regions.^[^ ^100^ ^]^

MPS is powerful in that it allows real‐time observation of drug effects on cell and tissue interactions in in vivo‐like microenvironments. For instance, the cytotoxic interplay between natural killer (NK) and cancer cells was monitored in a 3D environment over time.^[^ ^52^ ^]^ Recent advancements presented real‐time monitoring of NK cell extravasation, killing, and intravasation using MPS (Figure 3E).^[^ ^101^ ^]^ This real‐time assessment offers the advantage of evaluating diverse microenvironmental factors' effects, such as cancer cell type, cell concentration, and ECM composition,^[^ ^102^, ^103^, ^104^ ^]^ a critical aspect in understanding the role of MPS in cancer treatment.

Given the intricate nature of physiological processes involving interactions between multiple organs, MPS has embraced modeling multiorgan interactions for deeper insights into disease biology and drug responses. A multi‐organ chip, incorporating four distinct tissues (heart, liver, bone, and skin) cultured for over 4 weeks, has been developed to fully manifest molecular, structural, and functional phenotypes (Figure 3F).^[^ ^105^ ^]^ The interconnections between these mature tissues, facilitated by circulating a medium, allow efficient crosstalk among the organs. Notably, just 2 weeks into co‐culture, the presence of CD63 exosomes, secreted by heart tissues, was observed in all interconnected tissues, underscoring the dynamic communication and exchange of molecular signals within a multi‐organ chip system. Leveraging the unique features of patient samples, these models present a promising avenue for accurate and personalized evaluations in non‐clinical settings.

## 5 Developing Patient‐Derived Microphysiological Systems

As previously discussed, MPS has effectively mirrored the microphysiological intricacies of the human body, leveraging both cell lines and immortalized counterparts. Extending this paradigm, researchers are now venturing into the niche of patient‐specific in vitro models, harnessing the potential of patient‐derived specimens. The exploration of these specimens, ranging from cells directly isolated from patients to iPSCs and organoids, unveils a multifaceted landscape for precision medicine. Directly obtained patient cells provide a window into individual physiological variations, nurturing personalized in vitro models.^[^ ^106^ ^]^ On the other hand, iPSCs, reprogrammed from patient cells, broaden their clinical potential by serving as a versatile platform for generating diverse cell types that faithfully mimic the patient's biology.^[^ ^107^ ^]^ However, the intricacies and time demands of the reprogramming process pose a notable drawback.^[^ ^108^ ^]^ Organoids, dynamic 3D architecture cultured from patient cells, combine the benefits of individuality and complexity, faithfully replicating the structure and function of specific organs.^[^ ^109^ ^]^ Despite the considerable potential of organoids, challenges persist in terms of standardization and fully capturing the complexity of in vivo tissue (**Table** **1**). Here, we lay out a strategic approach for developing P‐MPS that optimally leverages the strengths and addresses the limitations associated with distinct patient‐derived samples (**Table** **2**).

**Table 1:** Comparative analysis of patient‐derived specimens.

Model	Definition	Clinical potential	Drawbacks	Ref
Patient‐derived cells (PDC)	Cells isolated directly from a patient, retaining the genetic makeup and physiological characteristics of the donor.	Patient individuality: Enables tailoring treatments based on individual patient characteristics.	Limited proliferation Heterogeneity Ethical considerations	[106, 110, 111]
Patient‐derived organoids (PDO)	Self‐organizing 3D structures derived from stem cells that mimic specific organs' structural and functional properties.	Organ‐specific functionality: Recapitulates organ‐like structures and functions, providing a more realistic model.	Complexity Limited vascularization Maturation	[109, 112, 113]
Induced Pluripotent Stem Cells (iPSC)	Reprogrammed cells that regress to a pluripotent state, similar to embryonic stem cells, derived from a patient's somatic cells.	Pluripotency: iPSCs can differentiate into various cell types, mirroring the diversity of the patient microenvironment	Genetic instability Tumorigenic potential Efficiency and reproducibility	[107, 108, 114]

**Table 2:** Comprehensive analysis of P‐MPS for patient‐derived cells (PDCs), organoids (PDOs), and iPSCs.

P‐MPS	Target	Resource	MPS features	Applications	Ref
PDC	Gastric	– Patient‐derived gastric cancer cell (Biopsy) – HUVEC and LF	‐ Injection‐molded plastic chip for vascularized spheroid culture	– Functional mimicry – Drug testing	[93]
	Gut	– Patient‐derived intestinal subepithelial myofibroblasts (Surgical resection and biopsy) – Endothelial colony forming cell‐derived endothelial cell (Umbilical cord blood) – Patient‐derived human intestinal epithelial cells (Biopsy)	– Channel‐over‐channel 2‐layer structure – Porous membrane between the channel – Perfused vasculature by gravity‐driven flow	– Functional mimicry	[94]
	Brain	– Patient‐derived glioma stem‐like cells (Biobank)	– Quasi‐cylindrical channel for microvessel – Cell‐gell interface along with the gravity‐driven flow	– Functional mimicry	[95]
	Breast	– Patient‐derived mammary cells	– Open fluidic chamber with removable PCL‐based electrospun scaffolds	– Functional mimicry	[96]
	Breast	– Patient‐derived breast circulating tumor cells (Blood)	– Multilayered microfluidic device – Polydimethylsiloxane layer (top) – Cyclic olefin copolymer layer(bottom)	– Cell sorting – Drug testing	[106]
	Prostate	– Prostate cancer (PCa) PDX cells (Biopsy)	– Commercially available multiwell chip with chemically modified gel	– Functional mimicry – Drug testing	[109]
PDO	Pancreas	– Patient‐derived pancreatic adenocarcinoma organoids (Biobank)	– Polystyrene open‐well multi‐channel microfluidic chip with gravity‐driven fluid flow	– Functional mimicry	[128]
	Pancreas	– Patient‐derived pancreatic ductal epithelial cells (Surgical resection) – Patient‐derived pancreatic islet cells (Surgical resection) – Patient‐derived acinar cells (Surgical resection)	– Two‐cell culture chambers with porous membrane for co‐culture of epithelium and islet cells	– Functional mimicry – Disease modeling	[129]
	Pancreas	– ‐Patient‐derived pancreatic organoids (Biobank) – Human dermal fibroblasts – Human endothelial cells	– The lumen of the tubular scaffold with HUVEC around the Matrigel encapsulated organoids	– Functional mimicry – Disease modeling – Drug testing	[130]
	Pancreas	– Pancreatic ductal adenocarcinoma organoids (Biopsy under endoscopic ultrasound) – Human pancreatic stellate cells – Human monocyte	– Multichambered microfluidic device with a porous membrane – Continuous perfusion using a syringe pump	– Functional mimicry – Drug testing	[98]
	Brain	– Patient‐derived glioblastoma organoids (Biobank)	– Microfluidic device for droplet generation using a syringe pump	– Functional mimicry – Drug testing	[139]
	Brain	– Patient‐derived glioblastoma (Surgical resection)	– Self‐transformable cell‐culture insert arrays by temperature	– Functional mimicry – Drug testing	[140]
	Breast	– patient‐derived breast cancer organoids (Biobank) – Endothelial colony‐forming cell‐derived endothelial cells (Cord blood)	– Microvascular chamber with 2 parallel fluidic lines	– Functional mimicry – Drug testing	[141]
	Breast	– Primary breast cancer tissue (Surgical resection)	– Microwell‐based arrays	– Functional mimicry – Drug testing	[142]
	Intestine	– Human intestinal organoids or tissue samples from patients (Surgical resection and Endoscopic biopsy) – ‐Microbiome from a healthy donor	– Two‐layered microfluidic device with a porous membrane	– Functional mimicry – Disease modeling	[153]
	Intestine	– Patient‐derived gastrointestinal cancer tumor	– Injection‐molded 96 units nested design array consists of a reservoir and a 3D implanting hole.	– Functional mimicry – Drug testing	[154]
	Lung	– Metastatic lung cancer organoids (Surgical resection and Biopsy)	– Microwell array chip	– Functional mimicry – Drug testing	[158]
iPSC	Blood–brain barrier	– Astrocyte – Pericyte – BMEC	– Membrane‐based bilayer cell culture microchannels	– Functional mimicry – Disease modeling – Drug testing	[25]
	Blood vessel	– Endothelial cell (HHT1) – Primary human brain vascular pericytes	– 3D co‐culture channel within micropost arrays	– Disease modeling – Functional mimicry	[165]
	Brain	– GABAergic neuron, Astrocyte	– Microfluidics‐based cell‐gel matrices and perfusion lanes	– Disease modeling – Drug testing	[167]
	Heart	– Cardiomyocytes – Vascular endothelial cells – Vascular mural cells	– Microchamber for cardiac microtissue – Diaphragm and push‐bar for beating motion	– Functional mimicry – Drug testing	[168]
	Pancreas	– Pancreatic progenitors	– Microwell chip to form 3D aggregates	– Functional mimicry	[169]
	Retina	– Retinal organoid – Retinal pigment epithelia (RPE)	– Organoid and RPE co‐culture chambers – Vascular‐like perfusion channels	– Functional mimicry – Disease modeling – Drug testing	[170]

### 5.1 PDC‐Based MPS

MPS that employ PDC is crucial for advancing personalized medicine and drug discovery, surpassing traditional in vitro models that rely on immortalized cell lines. PDCs possess personalized genetic characteristics and disease information, making them ideal representations of disease heterogeneity and individualized treatment responses.^[^ ^106^ ^]^ Compared with conventional cell lines, PDCs capture molecular properties and diseases more accurately in terms of genomic and transcriptomic profiling.^[^ ^115^ ^]^ PDCs are valuable tools for investigating cell‐to‐cell interactions,^[^ ^116^ ^]^ the influence of the microenvironment on disease progression,^[^ ^117^, ^118^ ^]^ and drug effectiveness.^[^ ^119^, ^120^, ^121^ ^]^

**Figure 4:**
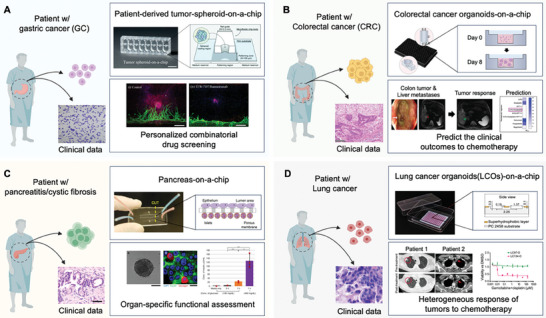
Representative examples of patient‐derived cell/organoid‐based MPS. A) A patient‐derived gastric cancer spheroid‐on‐a‐chip model for demonstrating blood vessel formation induced by PDC and evaluating the anti‐cancer drug efficacy. Green: HUVEC (lectin, 488), Red: Patient‐derived gastric cancer cell (EpCAM, 594). Reproduced with permission from Lee et al. (Cancer Medicine 10, 20 (2021); Copyright 2021 John Wiley & Sons Ltd.).^[^ ^123^ ^]^ B) A patient‐derived pancreas‐on‐a‐chip for mimicking defective conductance regulator (CFTR) function associated with cystic fibrosis. Defective CFTR function in pancreatic ductal epithelial cells (PDECs) led to a significant reduction in insulin secretion within islet cells. Reproduced with permission from Mun et al. (Nature Communications 10, 1 (2019); Copyright 2019, Springer Nature).^[^ ^139^ ^]^ C) A patient‐derived colorectal cancer organoid model on a nested array chip for high‐throughput drug screening. PDO model on the chip accurately predicted chemotherapy outcomes. Reproduced with permission from Cui et al. (Bio‐Design and Manufacturing 5 (2022); Copyright 2022 Springer).^[^ ^140^ ^]^ D) A superhydrophobic microwell array chip for patient‐derived lung cancer organoid models that recapitulate the histological and genetic features of the parental tumors to produce clinically meaningful drug responses within a week. Reproduced with permission from Hu et al. (Nature Communications 12,1 (2021); Copyright 2021, Springer Nature).^[^ ^141^ ^]^ Illustrations were created using BioRender.

In the context of tumor research, culturing patient‐derived tumor cells in a microfluidic chip allows high‐throughput drug screening and mimics TME.^[^ ^122^ ^]^ Lee et al. conducted a study on a cohort of patients with gastric cancer and provided pathological evidence.^[^ ^123^ ^]^ They utilized an MPS model to investigate the impact of high epithelial‐mesenchymal transition (EMT) on tumor invasiveness and angiogenesis. In the MPS model, they observed that spheroids from PDCs displayed spreading behavior and underwent angiogenesis facilitated by endothelial cells. Moreover, their findings indicated that a combination therapy involving the transforming growth factor‐β (TGF‐β) inhibitor TEW‐7197 and the antiangiogenic agent Ramucirumab could potentially yield benefits for patients exhibiting EMT (**Figure** **4**A).^[^ ^123^ ^]^ Seiler et al. developed a gut‐on‐a‐chip to create a model that faithfully replicated the architecture and functionality of the small intestine using small intestinal myofibroblasts obtained from patients.^[^ ^124^ ^]^ The use of a microfluidic device was particularly effective in generating the fluid flow and interstitial pressure induced by the vasculature in vivo. By incorporating patient‐derived myofibroblasts and endothelial cells, the model accurately recapitulates the cellular interactions and signaling pathways involved in capillary development in the small intestine. These findings indicate the role of myofibroblasts in directing capillary network formation and maintaining physiological responsiveness, emphasizing how variations in myofibroblast function affect capillary functionality. These insights have implications for personalized treatment strategies and the study of specific patient populations including those with inflammatory bowel disease and intestinal vascular disorders.

Culturing PDCs in MPS is also valuable for studying glioblastoma multiforme (GBM), an aggressive brain tumor well‐known for its cellular heterogeneity and resistance to therapy.^[^ ^125^ ^]^

This inherent heterogeneity poses significant challenges to the development of effective therapeutic strategies, as treatment approaches must account for various subpopulations of cancer cells with distinct properties and drug sensitivities.^[^ ^126^ ^]^ In MPS, the researchers observed the molecular interactions between patient‐derived perivascular niches and GBM cells.^[^ ^127^ ^]^ These interactions have been shown to regulate GBM migration, proliferation, and mesenchymal transition by upregulating the expression of vascular‐enriched integrin‐binding sialoprotein (IBSP). Moreover, investigating the cultivation of glioma cells within a perivascular niche‐like environment in the MPS revealed that it helps preserve their stem cell‐like properties.^[^ ^125^ ^]^ This approach provides controlled and reproducible conditions for studying glioma cell behavior and response to treatments, allowing for the exploration of essential microenvironment‐related signaling pathways, molecular crosstalk, and cellular behaviors contributing to glioma stemness and therapy resistance. In a similar way, co‐culturing glioma cells with endothelial cells has revealed increased migration toward the endothelial barrier and the upregulation of neovascularization‐related genes—key features of glioblastoma progression. The expression levels of neovascularization‐ and stemness‐related genes were compared between patient‐derived GBM cells and the conventional U87 human GBM cell line in both 2D and 3D cultures. Notably, only the PDC lines exhibited an upregulation of epidermal growth factor receptor (EGFR) expression. Furthermore, 3D collagen culture enhanced the expression of stemness‐related markers (CD44, CD133, nestin, oligo2, and sox2) and decreased proliferation (Ki67 marker) compared to 2D culture, suggesting that 3D collagen culture in MPS is beneficial for maintaining their phenotype. These systems aim to recreate the structural, functional, and cellular characteristics of specific organs or tissues using PDCs.^[^ ^19^ ^]^ PDCs harbor unique genetic mutations that provide valuable insights into disease characteristics and potential drug targets.^[^ ^128^ ^]^

PDCs offer a more accurate representation of in vivo conditions. It is noteworthy that even in the same experimental conditions, PDCs and cell lines often yield markedly different results. The patient‐derived mammary cells were cultured on chemically modified electrospun scaffolds within an open microfluidic system to create a biomimetic 3D environment resembling the in vivo ECM.^[^ ^129^ ^]^ Unlike mammary cell lines, newly obtained patient‐derived mammary cells require specialized surface coatings for adhesion and differentiation. Similarly, Piscitelli et al. also investigated the effect of surface oxidation through NaOH treatment on cell adhesion, proliferation, and differentiation using qRT‐PCR expression analysis of luminal markers in both PDC and cancerous cell lines.^[^ ^130^ ^]^ These MPS offer advantages including the ability to investigate disease mechanisms at the cellular level, evaluate drug responses, and provide scalability for various cell types and tissues.

Additionally, the use of PDC‐based MPS in combination with circulating tumor cells (CTCs) offers several advantages over traditional preclinical models. Recently, the use of liquid biopsy to obtain CTCs from patients has increased owing to its minimally invasive nature, accessibility, and cost‐effectiveness in diagnosing diseases and evaluating treatment efficacy.^[^ ^131^ ^]^ Even more, CTCs can be obtained from patients with various types of cancer including breast,^[^ ^132^ ^]^ colon,^[^ ^133^ ^]^ lung,^[^ ^134^ ^]^ ovarian,^[^ ^135^ ^]^ and prostate cancer.^[^ ^136^ ^]^ It allows the evaluation of patient‐specific responses to drugs, enabling a personalized approach to drug selection. This has the potential to significantly enhance treatment outcomes by identifying the most effective drug treatment regimens for individual patients, thereby minimizing unnecessary treatment‐related toxicities and improving the overall quality of patient care.^[^ ^137^ ^]^

On the other hand, the PDX model can be integrated into MPS to retain the patient's genetic information while increasing the number of cells for HTS or high‐throughput assays. Prostate cancer (PCa)‐derived PDX models were established in commercialized high‐throughput MPS using a chemically modified hyaluronic acid hydrogel.^[^ ^138^ ^]^ By co‐culturing PCa PDX with stromal fibroblasts and the endothelium in MPS, they enabled HTS and high‐content screening (HCS) and recapitulated aspects of the PCa TME in vitro. The specimens in this study were collected considering racial demographics, focusing on the well‐known gap in the incidence of PCa and overall survival between African American and European American men. These results demonstrate the utility of PDX‐based MPS for elucidating the underlying pathogenesis of cancer and determining drug treatments tailored to the unique requirements of diverse racial and ethnic groups. Overall, implementing PDX in MPS enables HTS/HCS of drug compounds and further detailed analysis. We believe that this approach allows researchers to study the impact of genetic variations, disease‐specific mutations, and personalized drug responses, leading to more effective treatments tailored to individual patients.

### 5.2 PDO‐Based MPS

PDOs can be established from patient specimens through biopsy or surgically resected specimens.^[^ ^142^ ^]^ The resected tissue was cultured and differentiated in ECM substances such as Matrigel or Basement membrane extract.^[^ ^143^ ^]^ PDO offers faster, more robust outcomes, accurately representing human tissue by recapitulating histopathologic profiles while maintaining genetic profiles across passaging.^[^ ^144^, ^145^ ^]^ The use of PDOs allows the creation of a personalized model of a patient's disease that can be used to validate potential drug treatments and identify effective therapies.^[^ ^146^ ^]^

While PDO exhibits substantial utilization on its own, its incorporation into an MPS platform significantly enhances physiological relevance. MPS enables researchers to replicate the intricate microenvironment surrounding organoids, thereby facilitating the accurate simulation of essential physiological conditions such as fluid flow, nutrient supply, and exposure to drugs or toxins. These exquisitely controlled conditions closely mimic the dynamic in vivo environment, which is essential for advancing our understanding of complex biological processes and drug responses. Thus, the integration of PDO with MPS enables the generation of highly physiologically relevant microenvironments, fostering more realistic and meaningful research in PDO. Herein, we introduce studies on PDO in diverse organs using a microfluidic platform.

PDO‐based MPS is extensively used in digestive system research. PDO‐based MPS offers a valuable platform for studying the intricate dynamics and functionality of the digestive system by replicating the complex cellular components and structural features of the gastrointestinal tract. With the use of MPS in digestive system biology, a comprehensive understanding of epithelial barrier function,^[^ ^147^, ^148^ ^]^ nutrient absorption,^[^ ^149^, ^150^ ^]^ interactions between gut microbiota,^[^ ^151^, ^152^, ^153^ ^]^ and drug metabolism^[^ ^152^, ^154^ ^]^ can be achieved by incorporating multiple cell types such as enterocytes, goblet cells, and immune cells.

A 3D physiodynamic mucosal interface was recreated in MPS for modeling microbiome‐associated diseases using biopsy‐derived organoids from patients with Crohn's disease, ulcerative colitis, and colorectal cancer (CRC).^[^ ^155^ ^]^ This involved mimicking the lumen and capillary compartments of the gut through 3D regeneration of the intestinal epithelium. In particular, the co‐culture of PDOs with the human fecal microbiome at the anoxic‐oxic interface has revealed the intricate dynamics of patient‐specific pathophysiology and the complex interplay between the host and microbiome. Most recently, the gastric mucosal defense system of the human stomach was successfully recapitulated through the combination of gastric organoids and MPS technology.^[^ ^156^ ^]^ In this study, the generation of fluid flow in MPS was a key factor in epithelial‐mesenchymal interaction, allowing functional maturation of gastric epithelial cells. As a result, they faithfully mimicked mesh‐like mucus layers containing a high level of mucus‐protective peptides, and epithelial junctional complexes were successfully reproduced, demonstrating their close similarity to natural biological structures. Furthermore, the nested array chip has effectively demonstrated the facilitation of a PDO model from CRC tissue obtained from three different patients, indicating its capability for high‐throughput drug screening (Figure 4B).^[^ ^140^ ^]^ They assessed the drug sensitivity of organoids to nine different treatment regimens and confirmed the heterogeneous response among patients and a possible correlation with clinical outcomes based on enhanced computerized tomography and magnetic resonance imaging. Similarly, Ooft et al. established the culturing of PDOs from patients with metastatic CRC in MPS to assess the efficacy of irinotecan‐based chemotherapy.^[^ ^157^ ^]^ As a result, more than 80% of patients were correctly identified as responders to treatment, avoiding the misclassification of individuals who would have benefited from this therapeutic approach. Based on several studies, including those above, it is evident that the key feature of the PDO model is its higher genetic similarity to the original patient tumors and its response to targeted drugs, exhibiting both intra‐ and inter‐patient heterogeneity.^[^ ^115^, ^158^, ^159^, ^160^ ^]^

Pancreas is a crucial organ in digestion, absorption, as well as the utilization and storage of energy substrates. On the other hand, pancreatic ductal adenocarcinoma (PDAC) stands as the most common type of pancreatic cancer worldwide.^[^ ^161^ ^]^ The microenvironment of PDAC tumors was replicated by co‐culturing patient‐derived pancreatic organoids with human fibroblasts and endothelial cells, utilizing a synthetic polymeric elastomer scaffold, as demonstrated.^[^ ^162^ ^]^ Within this model, it was observed that the presence of stromal components in the TME led to the increased size of pancreatic cancer organoids, encouraged pro‐tumorigenic immune cell activity, and stimulated collagen deposition around the tumor.^[^ ^139^, ^163^ ^]^ Likewise, Lai et al. also observed that the incorporation of stromal fibroblasts around the organoids resulted in increased collagen deposition, enhanced tissue elasticity, and elevated levels of cytokines and growth factors including interleukin‐6 (IL‐6), monocyte chemoattractant protein‐1, and TGF‐ß1.^[^ ^163^ ^]^ Particularly, TGF‐ß1 is a well‐known cytokine responsible for increasing type I collagen deposition in tumor stroma.^[^ ^164^ ^]^ They also observed the efficacy of gemcitabine, confirming that increased matrix stiffness acts as a physical barrier impeding small‐molecule transport through the vasculature. Another research also supports the results in terms of tumor progression, enhanced cancer‐associated gene expression, evasion of immune cells, and deposition of collagen.^[^ ^122^ ^]^ They also validated that TME‐modulating agents targeting stromal cells or macrophages increase the efficacy of chemotherapy. All these observations could only be achieved through the use of MPS, which was originally not possible with previous in vitro platforms.

On the other hand, cystic fibrosis (CF) is a genetic disorder caused by a defective gene that produces the cystic fibrosis transmembrane conductance regulator (CFTR).^[^ ^165^ ^]^ It is well known that people with CF can develop diabetes because of a glucose imbalance caused by impaired glucose metabolism. In this study, MPS played a pivotal role in emulating in vivo conditions within an in vitro platform. For instance, the faithful mimicking of cystic fibrosis‐related diabetes was achieved by generating organoids from isolated pancreatic ductal epithelial cells (PDECs) in the MPS.^[^ ^166^ ^]^ Additionally, a pancreas‐on‐a‐chip enabled the assessment of organ‐specific functions, revealing a 54% reduction in insulin secretion by islet cells due to attenuated CFTR function in PDECs (Figure 4C).^[^ ^139^ ^]^ These applications highlight the significant contribution of MPS in replicating and investigating in vivo phenomena within controlled laboratory settings.

The development of PDO on microfluidic platforms has emerged as a promising tool for studying the physiology and pathology of the reproductive system. It replicates the complex architecture of various reproductive organs such as the breast, ovaries, and prostates. The culture of patient‐derived breast tumor organoids cultured in MPS for several weeks revealed the hallmark features of tumor progression including angiogenesis and tumor cell intravasation.^[^ ^167^ ^]^ They also compared the angiogenic responses of breast cancer‐associated fibroblasts (CAFs) and normal fibroblasts (NFs) and found that proangiogenic factors, such as vascular endothelial growth factor (VEGF) and TGF‐β, were significantly increased in CAFs‐co‐cultured cases compared to NFs‐co‐cultured cases. Furthermore, a chemically cross‐linked biomimetic nanofibrillar hydrogel can be incorporated into a microfluidic array to improve culture conditions.^[^ ^143^ ^]^ Interestingly, tumor organoids cultured in the nanofibrillar hydrogel exhibited remarkable similarities to their parental tumors in terms of histopathological features, gene expression, and drug responses. Similar to this, 36 ovarian cancer (OC) organoids from 23 patients with OC, characterized at the whole‐genome level, were evaluated for their ability to preserve the genomic signature of the original tumor and accurately replicate patient responses to neoadjuvant carboplatin/paclitaxel combination therapy.^[^ ^160^ ^]^ The PDOs demonstrated significant heterogeneity in drug responses to targeted therapies within and between patients. These results were reproduced in neuroendocrine prostate cancer‐derived organoids. Further investigation confirmed that organoids derived from metastatic castration‐resistant adenocarcinoma tumors retain the genomic, transcriptomic, and epigenomic features of the corresponding patients over time and maintain similar responses to drugs in vitro.^[^ ^115^ ^]^ Those findings imply that PDO‐based MPS can serve as a powerful tool for precision medicine applications and personalized therapeutic approaches.

As mentioned in the PDC section, GBM is a highly aggressive and malignant brain tumor^[^ ^168^ ^]^ characterized by a highly heterogeneous nature, exhibiting diverse genetic and molecular profiles within tumors.^[^ ^169^, ^170^ ^]^ Compared to other organs, the brain is more challenging to study due to its limited accessibility, which hinders brain‐related research. For this reason, the integration of brain organoids into MPS helps overcome the limitations and contributes to the development of a reliable platform that enables extended studies of brain disease mechanisms. Jacob et al. cultured glioblastoma organoids (GBOs), preserving parental tumor cellular heterogeneity, histological features, gene expression, and mutational profiles.^[^ ^171^ ^]^ They demonstrated the utility of GBOs as a means of testing tailored therapies by evaluating the efficacy of specific drugs, such as trametinib or everolimus, and antigen‐specific chimeric antigen receptor T (CAR‐T) cell immunotherapy in a microfluidic chip. A 4D printed thermoresponsive shape memory polymer array was presented as a reliable and robust method for generating GBO and conducting histological analysis.^[^ ^172^ ^]^ By transforming cell culture inserts into histological cassettes upon heating, this array facilitated enhanced monitoring of tumor multicellular and clonal interactions. The method allowed for improved assessment of metabolic responses to therapy at the native tissue level with increased precision.

Furthermore, PDO‐based MPS serves as a versatile model capable of mimicking various organs, expanding its applicability. The head and neck squamous cell carcinoma organoids were subsequently used at MPS for chemotherapeutic screening in a semi‐automated manner.^[^ ^140^ ^]^ Lung cancer organoids (LCOs) and normal bronchial organoids from patient tissues were compared in MPS, revealing distinct histological subtypes of lung cancer and non‐neoplastic bronchial mucosa.^[^ ^159^ ^]^ Notably, LCO exhibited concordant responses to targeted drugs based on specific genomic alterations. Furthermore, Hu et al. shortened the LCO‐based drug sensitivity test period to 1 week using a superhydrophobic microwell array chip. They also succeeded in reducing the number of organoids required for testing and increasing the yield of organoid generation from a patient sample, making LCO‐based drug screening more feasible and realistic. In their study, the histological and genetic features of parental tumors were well reconstructed, and patient‐specific drug response prediction was highly correlated with clinical outcomes based on CT scan results. Furthermore, they confirmed the heterogeneous response of tumors to chemotherapy with cisplatin, gemcitabine, paclitaxel, and combination therapies (Figure 4D).^[^ ^141^ ^]^

The advantage of using a PDO‐based MPS in preclinical studies is that it conveys the genetic, epigenetic, and environmental factors of individual patients, resulting in more accurate disease modeling, detailed cellular analysis, evaluation of drug responses, and the development of tailored treatment approaches. It also enhances the understanding of human biology, enables precise therapeutic interventions, and holds great promise for advances in precision medicine.

### 5.3 Patient‐Derived iPSC‐Based MPS

**Figure 5:**
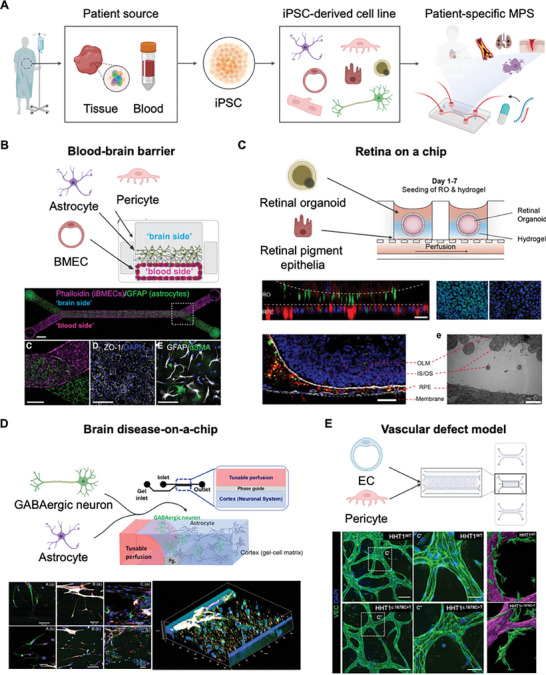
MPS with iPSCs developed from patient‐derived samples. A) The workflow involves obtaining iPSCs from a patient‐derived source and differentiating them into specific cell lines for the model. The P‐MPS facilitates the 3D co‐culture of two or more cell types, enabling a more comprehensive representation of cellular interactions. B) Development and validation of a personalized BBB model within the MPS. The BBB model was established by differentiating key cell lines (astrocyte, brain microvascular endothelial cell (BMEC), and pericyte) derived from iPSCs obtained from the same donor. Key protein expression was observed in each cell line from both the brain and blood sides of the membrane, indicating the functionality and relevance of the BBB model. Reproduced with permission from Vatine et al. (Cell stem cell 24, 6 (2019); Copyright 2019 Cell press).^[^ ^173^ ^]^ C) Microphysiological model of the human retina incorporating over seven essential retinal cell types derived from iPSCs. The retina on a chip cultures retinal pigment epithelia by introducing a downstream perfusion channel that can complement the avascularity of the retinal organoid. Reproduced with permission from Achberger et al. (Elife 8 (2019); Copyright 2019, eLife Sciences Publications Ltd.).^[^ ^174^ ^]^ D) 3D brain disease on chip platform with iPSC‐derived GABAergic neurons and astrocytes for studying neurotoxicity and therapeutic intervention in organophosphate exposure. Neuron‐astrocyte interaction, synapse formation, and enhanced viability with Butyrylcholinesterase treatment were observed. Reproduced with permission from Liu et al. (PLoS One 15, 3 (2020); Copyright 2022, PLOS).^[^ ^175^ ^]^ E) Patient‐specific hiPSCs were used to generate diseased and healthy endothelial cells reflecting haploinsufficiency. Characterization of the 3D vascular network in ECs with HHT gene mutations revealed abnormal morphological features, protein expression, and pericyte interaction. Reproduced with permission from Orlova et al. (Stem Cell Reports 17, 7 (2022); Copyright 2022, Cell Press).^[^ ^176^ ^]^ Illustrations were created using BioRender.

The use of iPSCs in P‐MPS offers several significant advantages. iPSCs possess remarkable plasticity, which enables them to differentiate into diverse cell types from single donors, thus facilitating the development of patient‐specific organ models with cells of the same origin.^[^ ^177^, ^178^ ^]^ These stem cells can be obtained from patient‐derived samples, such as blood or tissues, following a well‐defined differentiation protocol. Using these differentiated cells, a 3D cell culture was conducted within the MPS for the reconstruction of patient‐matched cells (**Figure** **5**A).^[^ ^179^ ^]^ Notably, all cells utilized in MPS are derived from patients, ensuring reliable disease modeling and an accurate representation of a patient's specific organ function.

The pluripotency of these iPSCs makes them ideal for BBB models that require a variety of cellular components. Derived from patients, these iPSCs were differentiated into brain microvascular endothelial‐like cells, astrocytes, and neurons, successfully establishing a barrier model within the MPS (Figure 5B).^[^ ^173^ ^]^ Through this P‐MPS, calcium imaging was employed to observe spontaneous neural activity, yielding physiologically relevant data. Notably, advancements in comprehending BBB function were achieved through assessments of brain function and transendothelial electrical resistance (TEER) measurements. Assessment of neural activity is also frequently utilized in muscle models, where iPSCs play a pivotal role in generating patient‐specific myocardium models.^[^ ^180^ ^]^ By developing cardiomyocytes and endothelial cells from iPSCs, researchers have created a spinal cord chip system to study human iPSCs. In this system, iPSCs were modeled as spinal cord neural progenitor cells and brain microvascular endothelial cells, providing a platform for validating neuronal activity through live calcium transient imaging.^[^ ^181^ ^]^ Researchers achieved enhanced alignment and ECM production in cardiac microtissues derived from genetically distinct hiPSC‐derived cardiomyocytes. The identification of a maturation medium rich in fatty acids marked a significant breakthrough, leading to improvements in mitochondrial structure, calcium handling, and action potential duration, and unveiling intriguing cell source‐dependent effects.^[^ ^182^ ^]^

Stem cell‐based models have paved the way for novel insights into structural and morphological aspects. Within P‐MPS, exocrine progenitors differentiated from iPSCs have successfully replicated ductal and acinar structures.^[^ ^183^ ^]^ Additionally, iPSC‐derived microphysiological models of the human retina, encompassing over seven crucial retinal cell types, have given rise to retinal organoids and retinal pigment epithelial cells in intricate 3D configurations (Figure 5C).^[^ ^174^ ^]^ To enhance the formation of outer segments in the retinal structure, a retina P‐MPS integrated iPSC‐derived retinal pigment epithelial cells, introducing downstream perfusion channels to complement the vascularity of retinal organoids. This reconstructed model, derived from patients with retinal degeneration, proved instrumental in assessing the potential for drug‐induced retinopathy.

Diseases caused by genetic variants in individual patients can be developed into excellent test beds when reconstituted in patient‐derived iPSCs. Recent studies have shown that iPSC‐derived GABA neurons rely on astrocytes for optimal growth and maturation (Figure 5D).^[^ ^176^ ^]^ In 3D brain‐on‐a‐chip models, a higher ratio of astrocytes to neurons improved cell viability after acute malathion exposure, consistent with previous findings on astrocyte resilience to neurite inhibition caused by diazinon. In addition, the use of 3D blood vessel models offers valuable insights into diseases, such as hemorrhagic telangiectasia, which arise from genetic mutations. The vascular defects were evaluated by assessing the leakiness and maturity of 3D microvessels (Figure 5E).^[^ ^184^ ^]^ To accomplish this, iPSCs encompassing both normal cells and cells with genetic mutations associated with hereditary hemorrhagic telangiectasia were generated. By inducing conditions that mimicked the disease state, they observed distinctive characteristics in the vascular networks developed from endothelial cells carrying genetic mutations. These vascular defects are absent in normal endothelial cells, emphasizing the significance of this 3D model using iPSC sources. The integration of gene‐editing technology with MPS offers a valuable approach to understanding the role of genetic variants in disease development. Sone et al. used patient‐derived iPSCs to compare genetically edited cells with gene point mutations associated with primary ciliary dyskinesia (PCD).^[^ ^175^ ^]^ A multicellular ciliopathy model was created by combining iPSCs with microfluidic airway‐on‐a‐chip technology. The airway chip, subjected to fluidic shear stress, allowed the quantification of ciliary motility as a key endpoint. Notably, the results demonstrated that gene‐corrected cells exhibited normal unidirectional fluid flow, in contrast to the impaired fluid flow observed in PCD cells. This integrated approach provides a platform for studying ciliopathies and exploring potential therapeutic interventions by providing insights into the functional impact of specific gene variants on cell behavior and disease phenotypes. These remarkable advancements extend beyond vascular‐related diseases, as iPSCs have been utilized to successfully develop various disease models including microfluidic airway‐on‐a‐chip,^[^ ^184^ ^]^ cardiac MPS,^[^ ^185^ ^]^ pancreatic duct models for cystic fibrosis,^[^ ^186^ ^]^ and retina‐on‐a‐chip platforms for modeling retinitis pigmentosa.^[^ ^174^ ^]^ By leveraging patient‐derived iPSCs in MPS, the field can effectively address challenges associated with inter‐patient variability and drug toxicity.

## 6 Perspective

### 6.1 Multicellular Co‐Culture of PDCs of Various Types

Recent studies have shown that TME plays a crucial role in cancer progression^[^ ^187^ ^]^ and drug resistance.^[^ ^188^, ^189^ ^]^ The TME comprises various cell types including stromal and immune cells and ECM components.^[^ ^190^, ^191^ ^]^ Interactions between cancer cells and the TME can promote cancer cell survival,^[^ ^192^ ^]^ invasion,^[^ ^193^ ^]^ metastasis,^[^ ^194^, ^195^ ^]^ immune cell evasion,^[^ ^196^, ^197^ ^]^ and increase resistance to therapy.^[^ ^198^, ^199^ ^]^ As a result, considering both tumor cells and TME could be a promising strategy for cancer therapy. Recent studies have identified novel components within the TME, such as CAFs and tumor‐associated macrophages (TAM), which can be targeted to improve treatment outcomes.^[^ ^200^, ^201^, ^202^ ^]^ Consequently, understanding the complex interactions within the TME may lead to the rapid selection of more effective combination therapies targeting multiple pathways involved in cancer progression. Therefore, to fully understand the complex interactions between tumor cells and the surrounding TME, it is necessary to obtain tumor and TME cells, including CAF and TAM, directly from a patient. Using this approach, it is possible to accurately replicate the unique characteristics of TME in each patient, providing valuable insights into the underlying biological mechanisms of tumorigenesis and potential therapeutic targets.

Similarly, over the past few decades, brain science research has shifted from studying only neurons to studying cells involved in brain inflammation, particularly astrocytes, microglia, and oligodendrocytes.^[^ ^203^, ^204^, ^205^ ^]^ This is important because it plays a crucial role in brain development and neurodegeneration.^[^ ^206^, ^207^ ^]^ Some patients may have mutations exclusively in astrocytes, others may have mutations in pericytes, and others may have mutations in all constituent cells.^[^ ^208^, ^209^ ^]^ As a result, a brain‐on‐a‐chip must be constructed from cells derived from the same individual, as each cell appears to be derived from a unique patient and exhibits unique characteristics. However, isolating individual cells from patient‐derived tissues, storing them, and creating libraries remain difficult and challenging to overcome.

### 6.2 In Vitro Modeling of the Human Immune System

Several diseases are associated with the immune system. Generally, these diseases are caused by immune system disorders or interactions between inflammatory and pathogenic cells. Rheumatoid arthritis (RA) is an autoimmune disease.^[^ ^210^ ^]^ It is a chronic inflammatory joint disease characterized by the destruction of bones and joints.^[^ ^211^ ^]^ In addition, multiple sclerosis (MS),^[^ ^212^ ^]^ Crohn's disease,^[^ ^213^ ^]^ and type 1 diabetes (T1D)^[^ ^214^ ^]^ are believed to be caused by autoimmune disorders; however, the exact cause is not yet fully understood.^[^ ^215^ ^]^ MS is an autoimmune disorder of the central nervous system characterized by inflammatory demyelination and axonal and neuronal injuries.^[^ ^216^ ^]^ Crohn's disease and T1D diabetes are T‐cell‐mediated autoimmune diseases.^[^ ^217^, ^218^ ^]^

Immunotherapy is a promising approach to cancer treatment. Unlike conventional cancer treatments, such as chemotherapy and radiation, which can have severe side effects, immunotherapy boosts the patient's immune system. These include engineering T cells to express chimeric antigen receptors, inactivating immune checkpoints (e.g., anti‐PD‐1 or anti‐CTLA‐4), dendritic cell‐based vaccines, and adoptive cell transfer therapy, all of which have shown remarkable clinical outcomes in certain cancers.^[^ ^219^, ^220^ ^]^ In recent years, CAR‐T cell therapy has been successful in treating hematological malignancies, such as leukemia and lymphoma, as well as solid tumors including lung cancer and melanoma.^[^ ^221^, ^222^ ^]^ Additionally, the immune system is closely associated with the etiology and treatment of various immune‐related diseases. Therefore, there is an increasing need for in vitro immune models. However, despite the advancements in this field, replicating the complexity of the human immune system in biomimetic models remains a major challenge. Consequently, there is a significant lack of reliable in vitro models that incorporate a patient‐specific immune system, whether overactive or weakened. Addressing this gap is crucial for advancing our understanding of immune‐related diseases and for developing personalized treatments that could significantly improve patient outcomes.

### 6.3 Clinical Translation of P‐MPS

The successful integration of P‐MPS technology into clinical and pharmaceutical applications hinges on the standardization of the generated data. P‐MPS not only excels in replicating intricate physiological environments but also ensures the reliability and uniformity of the resulting data. To achieve effective clinical translation, several key considerations must be addressed.

First, establishing a standardized experimental workflow is paramount including the pretreatment of patient samples introduced into the MPS.^[^ ^56^, ^223^, ^224^ ^]^ Creating frameworks within categories, such as PDC, iPSC, and organoid, will streamline the experimental process, enhancing accessibility for a broader user base. Second, a systematic assay methodology based on P‐MPS needs to be instituted. Establishing the experimental methodology will allow tailoring the assay according to the desired output or specific field requirements. For instance, criteria or indices can be devised to evaluate the real‐time efficiency of immune cells in killing cancer cells.^[^ ^52^, ^225^ ^]^ Barrier models like the BBB can guide the generation of standardized data from MPS equipped with sensors like TEER around the barrier.^[^ ^226^, ^227^ ^]^ Researchers can collaboratively develop and share assay methods, incorporating real‐time/functional data collection from P‐MPS and integrating sensors within the device.^[^ ^66^, ^228^ ^]^ Third, adopting AI‐driven processing for swift and reliable data generation is crucial, as previously discussed. Automation technologies, robotics, and high‐throughput MPS are advancing data collection, providing a wealth of information that can be harnessed to train powerful AI systems. These systems, well‐versed in extensive datasets, become adept at analyzing new data, yielding tools applicable in pharmaceutical and clinical settings.^[^ ^35^, ^229^, ^230^, ^231^, ^232^ ^]^ Finally, navigating the intricate regulatory landscape, especially with patient‐derived samples, demands adherence to regulatory requirements. Designing criteria and validation processes to correlate P‐MPS data with clinical trials for informed clinical decision‐making is essential. It may conduct supportive animal testing, bridging the gap between laboratory findings and real‐world applications.^[^ ^233^, ^234^ ^]^ Building strong collaborative networks is highlighted as a strategic imperative, emphasizing the collective effort required to propel P‐MPS from innovative technology to practical clinical and pharmaceutical integration.^[^ ^1^, ^235^, ^236^ ^]^

## 7 Conclusion

Recognition of the importance of patient‐centered diagnosis and treatment has highlighted the need for advanced nonclinical studies that can generate clinically relevant data to support precision medicine. MPS has now advanced to a stage where it can incorporate patient‐derived samples, including cells, tissues, and organoids, to create patient‐mimicking avatars. They served as physiologically relevant, high‐throughput platforms for conducting assays. By leveraging these advancements, a previously unmet clinical need for a patient‐centered nonclinical assessment platform may now be met, enabling more accurate and personalized approaches to medical research and drug development.

## Conflict of Interest

The authors declare no conflict of interest.

## Author Contributions

J.K. and J.S. contributed equally to this work. All the authors contributed to the writing, discussion, and editing of the manuscript.

## References

[1] C. Ma , Y. Peng , H. Li , W. Chen , Trends Pharmacol. Sci. 2021, 42, 119. 33341248 10.1016/j.tips.2020.11.009 PMC7990030

[2] A. Roth , Science 2021, 373, 1304. 34529479 10.1126/science.abc3734

[3] C. P. Miller , W. Shin , E. H. Ahn , H. J. Kim , D.‐H. Kim , Trends Biotechnol. 2020, 38, 857. 32673588 10.1016/j.tibtech.2020.01.003 PMC7368088

[4] A. A. Seyhan , Transl. Med. Commun. 2019, 4, 18.

[5] M. Crippa , S. Bersini , M. Gilardi , C. Arrigoni , S. Gamba , A. Falanga , C. Candrian , G. Dubini , M. Vanoni , M. Moretti , Lab Chip 2021, 21, 1061. 33522559 10.1039/d0lc01011a

[6] A. Selahi , T. Fernando , S. Chakraborty , M. Muthuchamy , D. C. Zawieja , A. Jain , Lab Chip 2022, 22, 121. 10.1039/d1lc00720c PMC9761984 34850797

[7] K. A. Homan , N. Gupta , K. T. Kroll , D. B. Kolesky , M. Skylar‐Scott , T. Miyoshi , D. Mau , M. T Valerius , T. Ferrante , J. V. Bonventre , J. A. Lewis , R. Morizane , Nat. Methods 2019, 16, 255. 30742039 10.1038/s41592-019-0325-y PMC6488032

[8] A. R. Baudy , M. A. Otieno , P. Hewitt , J. Gan , A. Roth , D. Keller , R. Sura , T. R. Van Vleet , W. R. Proctor , Lab Chip 2020, 20, 215. 31799979 10.1039/c9lc00768g

[9] C. P. Pires De Mello , C. Carmona‐Moran , C. W. Mcaleer , J. Perez , E. A. Coln , C. J. Long , C. Oleaga , A. Riu , R. Note , S. Teissier , J. Langer , J. J. Hickman , Lab Chip 2020, 20, 749. 31970354 10.1039/c9lc00861f PMC7123528

[10] S. Fowler , W. L. K. Chen , D. B. Duignan , A. Gupta , N. Hariparsad , J. R. Kenny , W. G Lai , J. Liras , J. A. Phillips , J. Gan , Lab Chip 2020, 20, 446. 31932816 10.1039/c9lc00857h

[11] K. M. Fabre , L. Delsing , R. Hicks , N. Colclough , D. C. Crowther , L. Ewart , Adv. Drug Delivery Rev. 2019, 140, 129. 10.1016/j.addr.2018.09.009 30253201

[12] V. Chokkalingam , J. Tel , F. Wimmers , X. Liu , S. Semenov , J. Thiele , C. G. Figdor , W. T. S. Huck , Lab Chip 2013, 13, 4740. 24185478 10.1039/c3lc50945a

[13] G. Sathyanarayanan , M. Haapala , T. Sikanen , Anal. Chem. 2020, 92, 14693. 33099994 10.1021/acs.analchem.0c03258

[14] A. W. J. Van Renterghem , J. Van De Haar , E. E. Voest , Nat. Rev. Clin. Oncol. 2023, 20, 305. 36914745 10.1038/s41571-023-00745-2

[15] A. Stewart , D. Denoyer , X. Gao , Y.‐C. Toh , Drug Discovery Today 2023, 28, 103496. 36690176 10.1016/j.drudis.2023.103496

[16] P. Malaney , S. V. Nicosia , V. Davé , Cancer Lett. 2014, 344, 1. 24157811 10.1016/j.canlet.2013.10.010 PMC4092874

[17] A. Gough , A. Soto‐Gutierrez , L. Vernetti , M. R. Ebrahimkhani , A. M. Stern , D. L. Taylor , Nat. Rev. Gastroenterol. Hepatol. 2021, 18, 252. 33335282 10.1038/s41575-020-00386-1 PMC9106093

[18] M. Campisi , S. E. Shelton , M. Chen , R. D. Kamm , D. A. Barbie , E. H. Knelson , Cancers 2022, 14, 3561. 35892819 10.3390/cancers14153561 PMC9330888

[19] U. Marx , T. Akabane , T. B. Andersson , E. Baker , M. Beilmann , S. Beken , S. Brendler‐Schwaab , M. Cirit , R. David , E.‐M. Dehne , ALTEX 2020, 37, 365. 32113184 10.14573/altex.2001241 PMC7863570

[20] H. N. Kim , N. L. Habbit , C.‐Y. Su , N. Choi , E. H. Ahn , E. A. Lipke , D.‐H. Kim , Adv. Funct. Mater. 2019, 29, 1807553.

[21] A. Moscona , Proc. Natl. Acad. Sci. U. S. A. 1957, 43, 184.16589996

[22] S. K. Rehman , J. Haynes , E. Collignon , K. R. Brown , Y. Wang , A. M. L. Nixon , J. P. Bruce , J. A. Wintersinger , A. Singh Mer , E. B. L. Lo , C. Leung , E. Lima‐Fernandes , N. M. Pedley , F. Soares , S. Mcgibbon , H. H. He , A. Pollet , T. J. Pugh , B. Haibe‐Kains , Q. Morris , M. Ramalho‐Santos , S. Goyal , J. Moffat , C. A. O'brien , Cell 2021, 184, 226. 33417860 10.1016/j.cell.2020.11.018 PMC8437243

[23] C. Krepler , K. Sproesser , P. Brafford , M. Beqiri , B. Garman , M. Xiao , B. Shannan , A. Watters , M. Perego , G. Zhang , A. Vultur , X. Yin , Q. Liu , I. N. Anastopoulos , B. Wubbenhorst , M. A. Wilson , W. Xu , G. Karakousis , M. Feldman , X. Xu , R. Amaravadi , T. C. Gangadhar , D. E. Elder , L. E. Haydu , J. A. Wargo , M. A. Davies , Y. Lu , G. B. Mills , D. T. Frederick , M. Barzily‐Rokni , et al., Cell Rep. 2017, 21, 1953. 29141225 10.1016/j.celrep.2017.10.021 PMC5726788

[24] J. H. Shin , J. H. Jung , H. Nam , S. W. Kim , D.‐W. Cho , G. Lim , BioChip J. 2015, 9, 67.

[25] E. Saland , H. Boutzen , R. Castellano , L. Pouyet , E. Griessinger , C. Larrue , F. de Toni , S. Scotland , M. David , G. Danet‐Desnoyers , F. Vergez , Y. Barreira , Y. Collette , C. Récher , J.‐E. Sarry , Blood Cancer J. 2015, 5, e297. 25794133 10.1038/bcj.2015.19 PMC4382660

[26] M. Rivera , I. Fichtner , A. Wulf‐Goldenberg , C. Sers , J. Merk , G. Patone , K. M. Alp , T. Kanashova , P. Mertins , J. Hoffmann , U. Stein , W. Walther , Neoplasia 2021, 23, 21. 33212364 10.1016/j.neo.2020.11.005 PMC7680704

[27] F. Reyal , C. Guyader , C. Decraene , C. Lucchesi , N. Auger , F. Assayag , L. De Plater , D. Gentien , M.‐F. Poupon , P. Cottu , P. De Cremoux , P. Gestraud , A. Vincent‐Salomon , J.‐J. Fontaine , S. Roman‐Roman , O. Delattre , D. Decaudin , E. Marangoni , Breast Cancer Res. 2012, 14, R11. 22247967 10.1186/bcr3095 PMC3496128

[28] M. M. Naldini , G. Casirati , M. Barcella , P. M. V. Rancoita , A. Cosentino , C. Caserta , F. Pavesi , E. Zonari , G. Desantis , D. Gilioli , M. G. Carrabba , L. Vago , M. Bernardi , R. Di Micco , C. Di Serio , I. Merelli , M. Volpin , E. Montini , F. Ciceri , B. Gentner , Nat. Commun. 2023, 14, 1285. 36890137 10.1038/s41467-023-36969-0 PMC9995364

[29] S.‐M. Kang , BioChip J. 2022, 16, 13.

[30] J. H. Sung , J. Koo , M. L. Shuler , BioChip J. 2019, 13, 115.

[31] M. Jang , H. N. Kim , BioChip J. 2023, 17, 133.

[32] C. M. Leung , P. De Haan , K. Ronaldson‐Bouchard , Ge‐Ah Kim , J. Ko , H. S. Rho , Z. Chen , P. Habibovic , N. Li Jeon , S. Takayama , M. L. Shuler , G. Vunjak‐Novakovic , O. Frey , E. Verpoorte , Yi‐C Toh , Nat. Rev. Methods Primers 2022, 2, 33.

[33] W. Zeng , L. Guo , S. Xu , J. Chen , J. Zhou , Trends Biotechnol. 2020, 38, 888. 32005372 10.1016/j.tibtech.2020.01.001

[34] Y. Wang , H. Jeon , Trends Pharmacol. Sci. 2022, 43, 569. 35504760 10.1016/j.tips.2022.03.014

[35] R. Novak , M. Ingram , S. Marquez , D. Das , A. Delahanty , A. Herland , B. M. Maoz , S. S. F. Jeanty , M. R. Somayaji , M. Burt , E. Calamari , A. Chalkiadaki , A. Cho , Y. Choe , D. B. Chou , M. Cronce , S. Dauth , T. Divic , J. Fernandez‐Alcon , T. Ferrante , J. Ferrier , E. A. Fitzgerald , R. Fleming , S. Jalili‐Firoozinezhad , T. Grevesse , J. A. Goss , T. Hamkins‐Indik , O. Henry , C. Hinojosa , T. Huffstater , et al., Nat. Biomed. Eng. 2020, 4, 407.31988458

[36] D.‐H. Choi , H.‐W. Liu , Y. H. Jung , J. Ahn , J.‐A. Kim , D. Oh , Y. Jeong , M. Kim , H. Yoon , B. Kang , E. Hong , E. Song , S. Chung , Lab Chip 2023, 23, 475. 36688448 10.1039/d2lc00983h

[37] J. Han , U. Kang , E.‐Y. Moon , H. Yoo , B. Gweon , BioChip J. 2022, 16, 255.

[38] K.‐I. Kamei , Y. Mashimo , Y. Koyama , C. Fockenberg , M. Nakashima , M. Nakajima , J. Li , Y. Chen , Biomed. Microdevices 2015, 17, 36. 25686903 10.1007/s10544-015-9928-y

[39] D. Lombardi , P. S. Dittrich , Expert Opin. Drug Discovery 2010, 5, 1081. 10.1517/17460441.2010.521149 22827746

[40] P. Vulto , S. Podszun , P. Meyer , C. Hermann , A. Manz , G. A. Urban , Lab Chip 2011, 11, 1596. 21394334 10.1039/c0lc00643b

[41] S. J. Trietsch , G. D. Israëls , J. Joore , T. Hankemeier , P. Vulto , Lab Chip 2013, 13, 3548. 23887749 10.1039/c3lc50210d

[42] F. Bonanini , D. Kurek , S. Previdi , A. Nicolas , D. Hendriks , S. De Ruiter , M. Meyer , M. Clapés Cabrer , R. Dinkelberg , S. B. García , B. Kramer , T. Olivier , H. Hu , C. López‐Iglesias , F. Schavemaker , E. Walinga , D. Dutta , K. Queiroz , K. Domansky , B. Ronden , J. Joore , H. L. Lanz , P. J. Peters , S. J. Trietsch , H. Clevers , P. Vulto , Angiogenesis 2022, 25, 455. 35704148 10.1007/s10456-022-09842-9 PMC9519670

[43] H. L. Lanz , A. Saleh , B. Kramer , J. Cairns , C. P. Ng , J. Yu , S. J. Trietsch , T. Hankemeier , J. Joore , P. Vulto , R. Weinshilboum , L. Wang , BMC Cancer 2017, 17, 709. 29096610 10.1186/s12885-017-3709-3 PMC5668957

[44] K. M. Bircsak , R. Debiasio , M. Miedel , A. Alsebahi , R. Reddinger , A. Saleh , T. Shun , L. A. Vernetti , A. Gough , Toxicology 2021, 450, 152667. 33359578 10.1016/j.tox.2020.152667

[45] B. P. Casavant , E. Berthier , A. B. Theberge , J. Berthier , S. I. Montanez‐Sauri , L. L. Bischel , K. Brakke , C. J. Hedman , W. Bushman , N. P. Keller , D. J. Beebe , Proc. Natl. Acad. Sci. U. S. A. 2013, 110, 10111. 23729815 10.1073/pnas.1302566110 PMC3690848

[46] E. Berthier , A. M. Dostie , U. N. Lee , J. Berthier , A. B. Theberge , Anal. Chem. 2019, 91, 8739. 31260266 10.1021/acs.analchem.9b01429 PMC7409765

[47] Y. Lee , J. W. Choi , J. Yu , D. Park , J. Ha , K. Son , S. Lee , M. Chung , H.‐Y. Kim , N. L. Jeon , Lab Chip 2018, 18, 2433. 29999064 10.1039/c8lc00336j

[48] S.‐R. Lee , S. Hyung , S. Bang , Y. Lee , J. Ko , S. Lee , H. J. Kim , N. L. Jeon , Biofabrication 2019, 11, 035013. 30917359 10.1088/1758-5090/ab1402

[49] S. Kim , J. Ko , S.‐R. Lee , D. Park , S. Park , N. L. Jeon , Biotechnol. Bioeng. 2021, 118, 2524. 33764506 10.1002/bit.27765

[50] N. Shin , Y. Kim , J. Ko , S. W. Choi , S. Hyung , S.‐E. Lee , S. Park , J. Song , N. L. Jeon , K.‐S. Kang , Biotechnol. Bioeng. 2022, 119, 566. 34716703 10.1002/bit.27978 PMC9298365

[51] J. Ko , J. Ahn , S. Kim , Y. Lee , J. Lee , D. Park , N. L. Jeon , Lab Chip 2019, 19, 2822. 31360969 10.1039/c9lc00140a

[52] D. Park , K. Son , Y. Hwang , J. Ko , Y. Lee , J. Doh , N. L. Jeon , Front. Immunol. 2019, 10, 1133. 31191524 10.3389/fimmu.2019.01133 PMC6546835

[53] Y.‐C. Tung , A. Y. Hsiao , S. G. Allen , Y.‐S. Torisawa , M. Ho , S. Takayama , Analyst 2011, 136, 473. 20967331 10.1039/c0an00609b PMC7454010

[54] Y. Kim , J. Ko , N. Shin , S. Park , S.‐R. Lee , S. Kim , J. Song , S. Lee , K.‐S. Kang , J. Lee , N. L. Jeon , Biotechnol. Bioeng. 2022, 119, 3678. 36043394 10.1002/bit.28221

[55] K. Tan , P. Keegan , M. Rogers , M. Lu , J. R. Gosset , J. Charest , S. S. Bale , Lab Chip 2019, 19, 1556. 30855604 10.1039/c8lc01262h

[56] M. Piergiovanni , S. B. Leite , R. Corvi , M. Whelan , Lab Chip 2021, 21, 2857. 34251386 10.1039/d1lc00241d

[57] L. Ewart , A. Roth , Nat. Rev. Drug Discovery 2021, 20, 327. 33619385 10.1038/d41573-020-00030-2

[58] A. Gerlach , G. Knebel , A. E. Guber , M. Heckele , D. Herrmann , A. Muslija , Th. Sshaller , Microsyst. Technol. 2002, 7, 265. 10.1515/bmte.2002.47.s1a.110 12451787

[59] J. Yu , S. Lee , J. Song , S.‐R. Lee , S. Kim , H. Choi , H. Kang , Y. Hwang , Y.‐K. Hong , N. L. Jeon , Nano Convergence 2022, 9, 16. 35394224 10.1186/s40580-022-00306-w PMC8994007

[60] M. Sahli , C. Millot , J.‐C. Gelin , T. Barrière , J. Mater. Process. Technol. 2013, 213, 913.

[61] V. N. Goral , Y.‐C. Hsieh , O. N. Petzold , R. A. Faris , P. K. Yuen , J. Micromech. Microeng. 2010, 21, 017002.

[62] U. N. Lee , X. Su , D. J. Guckenberger , A. M. Dostie , T. Zhang , E. Berthier , A. B. Theberge , Lab Chip 2018, 18, 496. 29309079 10.1039/c7lc01052d PMC5790604

[63] U. M. Attia , S. Marson , J. R. Alcock , Microfluid. Nanofluid. 2009, 7, 1.

[64] E. Macdonald , R. Wicker , Science 2016, 353, aaf2093. 27708075 10.1126/science.aaf2093

[65] S. Waheed , J. M. Cabot , N. P. Macdonald , T. Lewis , R. M. Guijt , B. Paull , M. C. Breadmore , Lab Chip 2016, 16, 1993. 27146365 10.1039/c6lc00284f

[66] H. Azizgolshani , J. R. Coppeta , E. M. Vedula , E. E. Marr , B. P. Cain , R. J. Luu , M. P. Lech , S. H. Kann , T. J. Mulhern , V. Tandon , K. Tan , N. J. Haroutunian , P. Keegan , M. Rogers , A. L. Gard , K. B. Baldwin , J. C. De Souza , B. C. Hoefler , S. S. Bale , L. B. Kratchman , A. Zorn , A. Patterson , E. S. Kim , T. A. Petrie , E. L. Wiellette , C. Williams , B. C. Isenberg , J. L. Charest , Lab Chip 2021, 21, 1454. 33881130 10.1039/d1lc00067e

[67] N. Brandenberg , S. Hoehnel , F. Kuttler , K. Homicsko , C. Ceroni , T. Ringel , N. Gjorevski , G. Schwank , G. Coukos , G. Turcatti , M. P. Lutolf , Nat. Biomed. Eng. 2020, 4, 863. 32514094 10.1038/s41551-020-0565-2

[68] C. A. Schneider , W. S. Rasband , K. W. Eliceiri , Nat. Methods 2012, 9, 671. 22930834 10.1038/nmeth.2089 PMC5554542

[69] A. E. Carpenter , T. R. Jones , M. R. Lamprecht , C. Clarke , In Kang , O. Friman , D. A. Guertin , J. Chang , R. A. Lindquist , J. Moffat , P. Golland , D. M. Sabatini , Genome Biol. 2006, 7, R100. 17076895 10.1186/gb-2006-7-10-r100 PMC1794559

[70] P. Henstock , Arch Pharmacol. Ther. 2021, 2, 24.

[71] M. I. Razzak , S. Naz , A. Zaib , in Classification in BioApps: Automation of Decision Making, Springer, New York 2018, pp.323–350.

[72] A. S. Ahuja , PeerJ 2019, 7, e7702. 31592346 10.7717/peerj.7702 PMC6779111

[73] J. Yoon , BioChip J. 2022, 16, 1. 10.1007/s13206-021-00044-x PMC8790009 35096279

[74] J. C. Kimmel , A. S. Brack , W. F. Marshall , IEEE/ACM Trans. Comput. Biol. Bioinf. 2019, 18, 562. 10.1109/TCBB.2019.2919307 31251191

[75] S. Lin , K. Schorpp , I. Rothenaigner , K. Hadian , Drug Discovery Today 2020, 25, 1348. 32561299 10.1016/j.drudis.2020.06.001

[76] Z. Chen , N. Ma , X. Sun , Q. Li , Yi Zeng , F. Chen , S. Sun , J. Xu , J. Zhang , H. Ye , J. Ge , Z. Zhang , X. Cui , K. Leong , Y. Chen , Z. Gu , Biomaterials 2021, 272, 120770. 33798957 10.1016/j.biomaterials.2021.120770

[77] L. Abdul , S. Rajasekar , D. S. Y. Lin , S. Venkatasubramania Raja , A. Sotra , Y. Feng , A. Liu , B. Zhang , Lab Chip 2020, 20, 4623. 33151236 10.1039/d0lc01010c

[78] T. Kassis , V. Hernandez‐Gordillo , R. Langer , L. G. Griffith , Sci. Rep. 2019, 9, 12479. 31462669 10.1038/s41598-019-48874-y PMC6713702

[79] Z. Ao , H. Cai , Z. Wu , L. Hu , A. Nunez , Z. Zhou , H. Liu , M. Bondesson , X. Lu , X. Lu , M. Dao , F. Guo , Proc. Natl. Acad. Sci. U. S. A. 2022, 119, e2214569119. 36343225 10.1073/pnas.2214569119 PMC9674214

[80] B. DoanNgan , D. Angus , L. Sung , BME Front. 2022, 2022, 9786242.37850170

[81] C. L. Chen , A. Mahjoubfar , L.‐C. Tai , I. K. Blaby , A. Huang , K. R. Niazi , B. Jalali , Sci. Rep. 2016, 6, 21471. 26975219 10.1038/srep21471 PMC4791545

[82] T. K. Matsui , Y. Tsuru , K. Hasegawa , K.‐I. Kuwako , Stem Cells 2021, 39, 1017. 33754425 10.1002/stem.3368

[83] S. Grebenyuk , A. Ranga , Front. Bioeng. Biotechnol. 2019, 7, 39. 30941347 10.3389/fbioe.2019.00039 PMC6433749

[84] D. Nothdurfter , C. Ploner , D. C. Coraça‐Huber , D. Wilflingseder , T. Müller , M. Hermann , J. Hagenbuchner , M. J. Ausserlechner , Biofabrication 2022, 14, 035002. 10.1088/1758-5090/ac5fb7 35333193

[85] V. S. Shirure , C. C. W. Hughes , S. C. George , Annu. Rev. Biomed. Eng. 2021, 23, 141. 33756087 10.1146/annurev-bioeng-090120-094330

[86] R. Michna , M. Gadde , A. Ozkan , M. Dewitt , M. Rylander , Biotechnol. Bioeng. 2018, 115, 2793. 29940072 10.1002/bit.26778 PMC6261298

[87] S. Kim , H. Lee , M. Chung , N. L. Jeon , Lab Chip 2013, 13, 1489. 23440068 10.1039/c3lc41320a

[88] J. Park , S. Kim , J. Hong , J. S. Jeon , Lab Chip 2022, 22, 4335. 36226506 10.1039/d2lc00597b

[89] Y. Nashimoto , R. Okada , S. Hanada , Y. Arima , K. Nishiyama , T. Miura , R. Yokokawa , Biomaterials 2020, 229, 119547. 31710953 10.1016/j.biomaterials.2019.119547

[90] V. Van Duinen , D. Zhu , C. Ramakers , A. J. Van Zonneveld , P. Vulto , T. Hankemeier , Angiogenesis 2019, 22, 157. 30171498 10.1007/s10456-018-9647-0 PMC6510881

[91] J. Ko , Y. Lee , S. Lee , S.‐R. Lee , N. L. Jeon , Adv. Healthcare Mater. 2019, 8, 1900328.

[92] M. Chung , S. Lee , B. J. Lee , K. Son , N. L. Jeon , J. H. Kim , Adv. Healthcare Mater. 2018, 7, 1700028. 10.1002/adhm.201700028 28557377

[93] D. E. Glaser , M. B. Curtis , P. A. Sariano , Z. A. Rollins , B. S. Shergill , A. Anand , A. M. Deely , V. S. Shirure , L. Anderson , J. M. Lowen , N. R. Ng , K. Weilbaecher , D. C. Link , S. C. George , Biomaterials 2022, 280, 121245. 34810038 10.1016/j.biomaterials.2021.121245 PMC10658812

[94] J. Ahn , J. Lim , N. Jusoh , J. Lee , T.‐E. Park , Y. Kim , J. Kim , N. L. Jeon , Front. Bioeng. Biotechnol. 2019, 7, 168. 31380359 10.3389/fbioe.2019.00168 PMC6653063

[95] N. Mori , Y. Morimoto , S. Takeuchi , Biomaterials 2017, 116, 48. 27914266 10.1016/j.biomaterials.2016.11.031

[96] B. S. Kim , Ge Gao , J. Y. Kim , D.‐W. Cho , Adv. Healthcare Mater. 2019, 8, 1801019.

[97] N. Jusoh , J. Ko , N. L. Jeon , APL Bioeng. 2019, 3, 036101. 31431937 10.1063/1.5093975 PMC6697035

[98] J. Seo , W. Y. Byun , F. Alisafaei , A. Georgescu , Y.‐S. Yi , M. Massaro‐Giordano , V. B. Shenoy , V. Lee , V. Y. Bunya , D. Huh , Nat. Med. 2019, 25, 1310. 31384041 10.1038/s41591-019-0531-2 PMC6950645

[99] S. Seo , S.‐Y. Nah , K. Lee , N. Choi , H. N. Kim , Adv. Funct. Mater. 2022, 32, 2106860.

[100] S. I. Ahn , Y. J. Sei , H‐Ji Park , J. Kim , Y. Ryu , J. J. Choi , H.‐J. Sung , T. J. Macdonald , A. I. Levey , Y. Kim , Nat. Commun. 2020, 11, 175. 31924752 10.1038/s41467-019-13896-7 PMC6954233

[101] J. Song , H. Choi , S. K. Koh , D. Park , J. Yu , H. Kang , Y. Kim , D. Cho , N. L. Jeon , Front. Immunol. 2021, 12, 733317. 34630415 10.3389/fimmu.2021.733317 PMC8500473

[102] N. Gopalakrishnan , R. Hannam , G. P. Casoni , D. Barriet , J. M. Ribe , M. Haug , Ø. Halaas , Lab Chip 2015, 15, 1481. 25608968 10.1039/c4lc01438c

[103] N. Subedi , L. C. Van Eyndhoven , A. M. Hokke , L. Houben , M. C. Van Turnhout , C. V. C. Bouten , K. Eyer , J. Tel , Sci. Rep. 2021, 11, 17084. 34429486 10.1038/s41598-021-96609-9 PMC8385055

[104] S. Parlato , G. Grisanti , G. Sinibaldi , G. Peruzzi , C. M. Casciola , L. Gabriele , Lab Chip 2021, 21, 234. 33315027 10.1039/d0lc00799d

[105] K. Ronaldson‐Bouchard , D. Teles , K. Yeager , D. N. Tavakol , Y. Zhao , A. Chramiec , S. Tagore , M. Summers , S. Stylianos , M. Tamargo , B. M. Lee , S. P. Halligan , E. H. Abaci , Z. Guo , J. Jacków , A. Pappalardo , J. Shih , R. K. Soni , S. Sonar , C. German , A. M. Christiano , A. Califano , K. K. Hirschi , C. S. Chen , A. Przekwas , G. Vunjak‐Novakovic , Nat. Biomed. Eng. 2022, 6, 351. 35478225 10.1038/s41551-022-00882-6 PMC9250010

[106] J.‐K. Lee , Z. Liu , J. K. Sa , S. Shin , J. Wang , M. Bordyuh , H. J. Cho , O. Elliott , T. Chu , S. W. Choi , D. I. S. Rosenbloom , I.‐H. Lee , Y. J. Shin , H. J. Kang , D. Kim , S. Y. Kim , M.‐H. Sim , J. Kim , T. Lee , Y. J. Seo , H. Shin , M. Lee , S. H. Kim , Y.‐J. Kwon , J.‐W. Oh , M. Song , M. Kim , D.‐S. Kong , J. W. Choi , H. J. Seol , et al., Nat. Genet. 2018, 50, 1399. 30262818 10.1038/s41588-018-0209-6 PMC8514738

[107] M. Bellin , M. C. Marchetto , F. H. Gage , C. L. Mummery , Nat. Rev. Mol. Cell Biol. 2012, 13, 713. 23034453 10.1038/nrm3448

[108] N. Sayed , C. Liu , J. C. Wu , J. Am. Coll. Cardiol. 2016, 67, 2161. 27151349 10.1016/j.jacc.2016.01.083 PMC5086255

[109] L. A. Baker , H. Tiriac , H. Clevers , D. A. Tuveson , Trends Cancer 2016, 2, 176. 27135056 10.1016/j.trecan.2016.03.004 PMC4847151

[110] D. P. Kodack , A. F. Farago , A. Dastur , M. A. Held , L. Dardaei , L. Friboulet , F. Von Flotow , L. J. Damon , D. Lee , M. Parks , R. Dicecca , M. Greenberg , K. E. Kattermann , A. K. Riley , F. J. Fintelmann , C. Rizzo , Z. Piotrowska , A. T. Shaw , J. F. Gainor , L. V. Sequist , M. J. Niederst , J. A. Engelman , C. H. Benes , Cell Rep. 2017, 21, 3298. 29241554 10.1016/j.celrep.2017.11.051 PMC5745232

[111] J. Y. Lee , S. Y. Kim , C. Park , N. K. D. Kim , J. Jang , K. Park , J. H. Yi , M. Hong , T. Ahn , O. Rath , J. Schueler , S. T. Kim , In‐Gu Do , S. Lee , S. H. Park , Y. I. Ji , D. Kim , J. O. Park , Y. S. Park , W. K. Kang , K.‐M. Kim , W.‐Y. Park , H. Y. Lim , J. Lee , OncoTargets Ther. 2015, 6, 25619. 10.18632/oncotarget.4627 PMC4694854 26296973

[112] S. Karkampouna , F. La Manna , A. Benjak , M. Kiener , M. De Menna , E. Zoni , J. Grosjean , I. Klima , A. Garofoli , M. Bolis , A. Vallerga , J.‐P. Theurillat , M. R. De Filippo , V. Genitsch , D. Keller , T. H. Booij , C. U. Stirnimann , K. Eng , A. Sboner , C. K. Y. Ng , S. Piscuoglio , P. C. Gray , M. Spahn , M. A. Rubin , G. N. Thalmann , M. Kruithof‐De Julio , Nat. Commun. 2021, 12, 1117. 33602919 10.1038/s41467-021-21300-6 PMC7892572

[113] M. Mebarki , A. Bennaceur , L. Bonhomme‐Faivre , Drug Discovery Today 2018, 23, 857. 29428171 10.1016/j.drudis.2018.02.003

[114] E. P. Papapetrou , Nat. Med. 2016, 22, 1392. 27923030 10.1038/nm.4238 PMC5233709

[115] L. Puca , R. Bareja , D. Prandi , R. Shaw , M. Benelli , W. R. Karthaus , J. Hess , M. Sigouros , A. Donoghue , M. Kossai , D. Gao , J. Cyrta , V. Sailer , A. Vosoughi , C. Pauli , Y. Churakova , C. Cheung , L. D. Deonarine , T. J. Mcnary , R. Rosati , S. T. Tagawa , D. M. Nanus , J. M. Mosquera , C. L. Sawyers , Y. Chen , G. Inghirami , R. A. Rao , C. Grandori , O. Elemento , A. Sboner , et al., Nat. Commun. 2018, 9, 2404. 29921838 10.1038/s41467-018-04495-z PMC6008438

[116] G. Piro , A. Agostini , A. Larghi , G. Quero , C. Carbone , A. Esposito , G. Rizzatti , F. Attili , S. Alfieri , G. Costamagna , G. Tortora , Front. Med. 2021, 8, 793144. 10.3389/fmed.2021.793144 PMC8733292 35004765

[117] M. C. Leiva , E. Garre , A. Gustafsson , A. Svanström , Y. Bogestål , J. Håkansson , A. Ståhlberg , G. Landberg , J. Cell. Physiol. 2021, 236, 4709. 33368325 10.1002/jcp.30191 PMC8049042

[118] A. A. Shafi , M. J. Schiewer , R. De Leeuw , E. Dylgjeri , P. A. Mccue , N. Shah , L. G. Gomella , C. D. Lallas , E. J. Trabulsi , M. M. Centenera , T. E. Hickey , L. M. Butler , G. V. Raj , W. D. Tilley , E. Cukierman , K. E. Knudsen , Eur. Urol. Oncol. 2018, 1, 325. 30467556 10.1016/j.euo.2018.04.019 PMC6241309

[119] S.‐Y. Cho , Lab. Anim. Res. 2020, 36, 14. 32461927 10.1186/s42826-020-00045-1 PMC7238616

[120] A. S. Ramalho , F. Amato , M. Gentzsch , J. Cystic Fibrosis 2022, 22, S32. 10.1016/j.jcf.2022.11.007 PMC9992303 36529661

[121] J. Jabs , F. M. Zickgraf , J. Park , S. Wagner , X. Jiang , K. Jechow , K. Kleinheinz , U. H. Toprak , M. A. Schneider , M. Meister , S. Spaich , M. Sütterlin , M. Schlesner , A. Trumpp , M. Sprick , R. Eils , C. Conrad , Mol. Syst. Biol. 2017, 13, 955. 29180611 10.15252/msb.20177697 PMC5731348

[122] M. R. Haque , C. R. Wessel , D. D. Leary , C. Wang , A. Bhushan , F. Bishehsari , Microsyst. Nanoeng. 2022, 8, 36. 35450328 10.1038/s41378-022-00370-6 PMC8971446

[123] S.‐Y. Lee , S. Byeon , J. Ko , S. Hyung , I.‐K. Lee , N. Li Jeon , J. Y. Hong , S. T. Kim , S. H. Park , J. Lee , Cancer Med. 2021, 10, 7253. 34542244 10.1002/cam4.4259 PMC8525100

[124] K. M. Seiler , A. Bajinting , D. M. Alvarado , M. A. Traore , M. M. Binkley , W. H. Goo , W. E. Lanik , J. Ou , U. Ismail , M. Iticovici , C. R. King , K. L. Vandussen , E. A. Swietlicki , V. Gazit , J. Guo , C. J. Luke , T. Stappenbeck , M. A. Ciorba , S. C. George , J. M Meacham , D. C. Rubin , M. Good , B. W. Warner , Sci. Rep. 2020, 10, 3842. 32123209 10.1038/s41598-020-60672-5 PMC7051952

[125] M. Gerigk , H. Bulstrode , H. H. Shi , F. Tönisen , C. Cerutti , G. Morrison , D. Rowitch , Y. Y. S. Huang , Lab Chip 2021, 21, 2343. 33969368 10.1039/d1lc00271f PMC8204159

[126] C. P. Couturier , S. Ayyadhury , P. U. Le , J. Nadaf , J. Monlong , G. Riva , R. Allache , S. Baig , X. Yan , M. Bourgey , C. Lee , Y. C. D. Wang , V. Wee Yong , M.‐C. Guiot , H. Najafabadi , B. Misic , J. Antel , G. Bourque , J. Ragoussis , K. Petrecca , Nat. Commun. 2020, 11, 3406. 32641768 10.1038/s41467-020-17186-5 PMC7343844

[127] Y. Ghochani , S. D. Muthukrishnan , A. Sohrabi , R. Kawaguchi , M. C. Condro , S. Bastola , F. Gao , Y. Qin , J. Mottahedeh , M. L. Iruela‐Arispe , Cell Rep. 2022, 41, 111511. 36261010 10.1016/j.celrep.2022.111511 PMC9642966

[128] A. S. Crystal , A. T. Shaw , L. V. Sequist , L. Friboulet , M. J. Niederst , E. L. Lockerman , R. L. Frias , J. F. Gainor , A. Amzallag , P. Greninger , D. Lee , A. Kalsy , M. Gomez‐Caraballo , L. Elamine , E. Howe , W. Hur , E. Lifshits , H. E. Robinson , R. Katayama , A. C. Faber , M. M. Awad , S. Ramaswamy , M. Mino‐Kenudson , A. J Iafrate , C. H. Benes , J. A. Engelman , Science 2014, 346, 1480. 25394791 10.1126/science.1254721 PMC4388482

[129] P. Guida , E. Piscitelli , M. Marrese , V. Martino , V. Cirillo , V. Guarino , E. Angeli , C. Cocola , P. Pelucchi , L. Repetto , ACS Biomater. Sci. Eng. 2020, 6, 3649. 33463182 10.1021/acsbiomaterials.0c00352

[130] E. Piscitelli , C. Cocola , F. R. Thaden , P. Pelucchi , B. Gray , G. Bertalot , A. Albertini , R. Reinbold , I. Zucchi , in Stem Cell Protocols, Springer, New York 2014, pp. 243–262. 10.1007/978-1-4939-1785-3_18 25388398

[131] A. Esposito , C. Criscitiello , M. Locatelli , M. Milano , G. Curigliano , Pharmacol. Ther. 2016, 157, 120. 26615782 10.1016/j.pharmthera.2015.11.007

[132] M. Yu , A. Bardia , N. Aceto , F. Bersani , M. W. Madden , M. C. Donaldson , R. Desai , H. Zhu , V. Comaills , Z. Zheng , B. S. Wittner , P. Stojanov , E. Brachtel , D. Sgroi , R. Kapur , T. Shioda , D. T. Ting , S. Ramaswamy , G. Getz , A. J Iafrate , C. Benes , M. Toner , S. Maheswaran , D. A. Haber , Science 2014, 345, 216. 25013076 10.1126/science.1253533 PMC4358808

[133] A. Soler , L. Cayrefourcq , T. Mazard , A. Babayan , P.‐J. Lamy , S. Assou , E. Assenat , K. Pantel , C. Alix‐Panabières , Sci. Rep. 2018, 8, 15931. 30374140 10.1038/s41598-018-34365-z PMC6206091

[134] G. Hamilton , O. Burghuber , R. Zeillinger , Lung 2015, 193, 451. 25821178 10.1007/s00408-015-9725-7

[135] R. Kar , D. Chawla , B. Gupta , M. Mehndiratta , N. Wadhwa , R. Agarwal , Int. J. Gynecol. Cancer 2017, 27, 2000. 28816710 10.1097/IGC.0000000000001087

[136] W. Liu , B. Yin , X. Wang , P. Yu , X. Duan , C. Liu , B. Wang , Z. Tao , Oncol. Lett. 2017, 14, 1223. 28789337 10.3892/ol.2017.6332 PMC5529747

[137] F. D. Schwab , M. C. Scheidmann , L. L. Ozimski , A. Kling , L. Armbrecht , T. Ryser , I. Krol , K. Strittmatter , B. D. Nguyen‐Sträuli , F. Jacob , Microsyst. Nanoeng. 2022, 8, 130. 36561926 10.1038/s41378-022-00467-y PMC9763115

[138] L. K. Sablatura , K. M. Bircsak , P. Shepherd , M. Bathina , K. Queiroz , M. C. Farach‐Carson , R. A. Kittles , P. E. Constantinou , A. Saleh , N. M. Navone , D. A. Harrington , Adv. Healthcare Mater. 2023, 12, 2201434. 10.1002/adhm.202201434 PMC10235208 36461624

[139] K. S. Mun , K. Arora , Y. Huang , F. Yang , S. Yarlagadda , Y. Ramananda , M. Abu‐El‐Haija , J. J. Palermo , B. N. Appakalai , J. D. Nathan , A. P. Naren , Nat. Commun. 2019, 10, 3124. 31311920 10.1038/s41467-019-11178-w PMC6635497

[140] Y. Cui , R. Xiao , Y. Zhou , J. Liu , Yi Wang , X. Yang , Z. Shen , B. Liang , K. Shen , Yi Li , G. Xiong , Y. Ye , X. Ai , Bio‐Des. Manuf. 2022, 5, 674.

[141] Y. Hu , X. Sui , F. Song , Y. Li , K. Li , Z. Chen , F. Yang , X. Chen , Y. Zhang , X. Wang , Q. Liu , C. Li , B. Zou , X. Chen , J. Wang , P. Liu , Nat. Commun. 2021, 12, 2581. 33972544 10.1038/s41467-021-22676-1 PMC8110811

[142] S. Mazzucchelli , F. Piccotti , R. Allevi , M. Truffi , L. Sorrentino , L. Russo , M. Agozzino , L. Signati , A. Bonizzi , L. Villani , F. Corsi , Biol. Proced. Online 2019, 21, 12. 31223292 10.1186/s12575-019-0099-8 PMC6570967

[143] E. Prince , J. Cruickshank , W. Ba‐Alawi , K. Hodgson , J. Haight , C. Tobin , A. Wakeman , A. Avoulov , V. Topolskaia , M. J. Elliott , A. P. Mcguigan , H. K. Berman , B. Haibe‐Kains , D. W. Cescon , E. Kumacheva , Nat. Commun. 2022, 13, 1466. 35304464 10.1038/s41467-022-28788-6 PMC8933543

[144] I. Romero‐Calvo , C. R. Weber , M. Ray , M. Brown , K. Kirby , R. K. Nandi , T. M. Long , S. M. Sparrow , A. Ugolkov , W. Qiang , Y. Zhang , T. Brunetti , H. Kindler , J. P. Segal , A. Rzhetsky , A. P. Mazar , M. M. Buschmann , R. Weichselbaum , K. Roggin , K. P. White , Mol. Cancer Res. 2019, 17, 70. 30171177 10.1158/1541-7786.MCR-18-0531 PMC6647028

[145] J. Kim , B.‐K. Koo , J. A. Knoblich , Nat. Rev. Mol. Cell Biol. 2020, 21, 571. 32636524 10.1038/s41580-020-0259-3 PMC7339799

[146] E. Driehuis , A. Van Hoeck , K. Moore , S. Kolders , H. E. Francies , M. C Gulersonmez , E. C. A. Stigter , B. Burgering , V. Geurts , A. Gracanin , G. Bounova , F. H. Morsink , R. Vries , S. Boj , J. Van Es , G. J. A. Offerhaus , O. Kranenburg , M. J. Garnett , L. Wessels , E. Cuppen , L. A. A. Brosens , H. Clevers , Proc. Natl. Acad. Sci. U. S. A. 2019, 116, 26580. 31818951 10.1073/pnas.1911273116 PMC6936689

[147] H. J. Kim , H. Li , J. J. Collins , D. E. Ingber , Proc. Natl. Acad. Sci. U. S. A. 2016, 113, E7. 26668389 10.1073/pnas.1522193112 PMC4711860

[148] H. J. Kim , D. Huh , G. Hamilton , D. E. Ingber , Lab Chip 2012, 12, 2165. 22434367 10.1039/c2lc40074j

[149] Y. Xiang , H. Wen , Y. Yu , M. Li , X. Fu , S. Huang , J. Tissue Eng. 2020, 11, 204173142096531. 10.1177/2041731420965318 PMC7682210 33282173

[150] Q. Ramadan , H. Jafarpoorchekab , C. Huang , P. Silacci , S. Carrara , G. Koklü , J. Ghaye , J. Ramsden , C. Ruffert , G. Vergeres , M. A. M. Gijs , Lab Chip 2013, 13, 196. 23184124 10.1039/c2lc40845g

[151] M. Maurer , M. S. Gresnigt , A. Last , T. Wollny , F. Berlinghof , R. Pospich , Z. Cseresnyes , A. Medyukhina , K. Graf , M. Gröger , M. Raasch , F. Siwczak , S. Nietzsche , I. D. Jacobsen , M. T. Figge , B. Hube , O. Huber , A. S. Mosig , Biomaterials 2019, 220, 119396. 31398556 10.1016/j.biomaterials.2019.119396

[152] W. Shin , H. J. Kim , Proc. Natl. Acad. Sci. U. S. A. 2018, 115, E10539. 30348765 10.1073/pnas.1810819115 PMC6233106

[153] J. Wu , B. Zhang , X. Liu , L. Peng , J. Liu , Y. Hu , X. Ji , H. Lv , S. Wang , Trends Food Sci. Technol. 2023, 134, 1.

[154] N. Milani , N. Parrott , D. Ortiz Franyuti , P. Godoy , A. Galetin , M. Gertz , S. Fowler , Lab Chip 2022, 22, 2853. 35833849 10.1039/d2lc00276k

[155] Y. C. Shin , W. Shin , D. Koh , A. Wu , Y. M. Ambrosini , S. Min , S. G Eckhardt , R. Y. D Fleming , S. Kim , S. Park , H. Koh , T. K. Yoo , H. J. Kim , Micromachines 2020, 11, 663. 32645991 10.3390/mi11070663 PMC7408321

[156] H.‐J. Jeong , Ji‐H Park , J. H. Kang , J. Sabaté Del Río , S‐Ho Kong , T.‐E. Park , Adv. Sci. 2023, 10, 2300164. 10.1002/advs.202300164 PMC10520631 37525340

[157] S. N. Ooft , F. Weeber , K. K. Dijkstra , C. M. Mclean , S. Kaing , E. Van Werkhoven , L. Schipper , L. Hoes , D. J. Vis , J. Van De Haar , W. Prevoo , P. Snaebjornsson , D. Van Der Velden , M. Klein , M. Chalabi , H. Boot , M. Van Leerdam , H. J. Bloemendal , L. V. Beerepoot , L. Wessels , E. Cuppen , H. Clevers , E. E. Voest , Sci. Transl. Med. 2019, 11, eaay2574. 31597751 10.1126/scitranslmed.aay2574

[158] G. Vlachogiannis , S. Hedayat , A. Vatsiou , Y. Jamin , J. Fernández‐Mateos , K. Khan , A. Lampis , K. Eason , I. Huntingford , R. Burke , M. Rata , D.‐M. Koh , N. Tunariu , D. Collins , S. Hulkki‐Wilson , C. Ragulan , I. Spiteri , S. Y. Moorcraft , I. Chau , S. Rao , D. Watkins , N. Fotiadis , M. Bali , M. Darvish‐Damavandi , H. Lote , Z. Eltahir , E. C. Smyth , R. Begum , P. A. Clarke , J. C. Hahne , et al., Science 2018, 359, 920. 29472484 10.1126/science.aao2774 PMC6112415

[159] M. Kim , H. Mun , C. O. Sung , E. J. Cho , H.‐J. Jeon , S.‐M. Chun , D. J. Jung , T. H. Shin , G. S. Jeong , D. K. Kim , E. K. Choi , S.‐Y. Jeong , A. M. Taylor , S. Jain , M. Meyerson , S. J. Jang , Nat. Commun. 2019, 10, 3991. 31488816 10.1038/s41467-019-11867-6 PMC6728380

[160] C. J. de Witte , J. E. Valle‐Inclan , N. Hami , K. Lõhmussaar , O. Kopper , C. P. H. Vreuls , G. N. Jonges , P. van Diest , L. Nguyen , H. Clevers , Cell Rep. 2020, 31, 107762. 32553164 10.1016/j.celrep.2020.107762

[161] P. Sarantis , E. Koustas , A. Papadimitropoulou , A. G. Papavassiliou , M. V. Karamouzis , World J. Gastrointest. Oncol. 2020, 12, 173. 32104548 10.4251/wjgo.v12.i2.173 PMC7031151

[162] B. F. L. Lai , R. X. Z. Lu , L. Davenport Huyer , S. Kakinoki , J. Yazbeck , E. Y. Wang , Q. Wu , B. Zhang , M. Radisic , Nat. Protoc. 2021, 16, 2158. 33790475 10.1038/s41596-020-00490-1

[163] B. F. L. Lai , R. X. Z. Lu , Y. Hu , L. Davenport Huyer , W. Dou , E. Y. Wang , N. Radulovich , M. S. Tsao , Y. Sun , M. Radisic , Adv. Funct. Mater. 2020, 30, 2000545. 33692660 10.1002/adfm.202000545 PMC7939064

[164] E. P. Amento , N. Ehsani , H. Palmer , P. Libby , Arterioscler. Thromb. 1991, 11, 1223. 1911708 10.1161/01.atv.11.5.1223

[165] N. Derichs , Eur. Respir. Rev. 2013, 22, 58. 23457166 10.1183/09059180.00008412 PMC9487424

[166] E. K. Schneider‐Futschik , Gene Ther. 2019, 26, 354. 31300729 10.1038/s41434-019-0092-5

[167] V. S. Shirure , Y. Bi , M. B. Curtis , A. Lezia , M. M. Goedegebuure , S. P Goedegebuure , R. Aft , R. C. Fields , S. C. George , Lab Chip 2018, 18, 3687. 30393802 10.1039/c8lc00596f PMC10644986

[168] A. C. Tan , D. M. Ashley , G. Y. López , M. Malinzak , H. S. Friedman , M. Khasraw , Ca‐Cancer J. Clin. 2020, 70, 299. 32478924 10.3322/caac.21613

[169] N. R. Parker , P. Khong , J. F. Parkinson , V. M. Howell , H. R. Wheeler , Front. Oncol. 2015, 5, 55. 25785247 10.3389/fonc.2015.00055 PMC4347445

[170] M.‐D.‐M. Inda , R. Bonavia , J. Seoane , Cancers 2014, 6, 226. 24473088 10.3390/cancers6010226 PMC3980595

[171] F. Jacob , R. D. Salinas , D. Y. Zhang , P. T. T. Nguyen , J. G. Schnoll , S. Z. H. Wong , R. Thokala , S. Sheikh , D. Saxena , S. Prokop , Di‐Ao Liu , X. Qian , D. Petrov , T. Lucas , H. I Chen , J. F. Dorsey , K. M. Christian , Z. A. Binder , M. Nasrallah , S. Brem , D. M. O'rourke , G‐Li Ming , H. Song , Cell 2020, 180, 188. 31883794 10.1016/j.cell.2019.11.036 PMC7556703

[172] M. Chadwick , C. Yang , L. Liu , C. M. Gamboa , K. Jara , H. Lee , H. E. Sabaawy , iScience 2020, 23, 101365. 32731171 10.1016/j.isci.2020.101365 PMC7393526

[173] G. D. Vatine , R. Barrile , M. J. Workman , S. Sances , B. K. Barriga , M. Rahnama , S. Barthakur , M. Kasendra , C. Lucchesi , J. Kerns , N. Wen , W. R. Spivia , Z. Chen , J. Van Eyk , C. N. Svendsen , Cell Stem Cell 2019, 24, 995. 31173718 10.1016/j.stem.2019.05.011

[174] K. Achberger , C. Probst , J. Haderspeck , S. Bolz , J. Rogal , J. Chuchuy , M. Nikolova , V. Cora , L. Antkowiak , W. Haq , Elife 2019, 8, e46188. 31451149 10.7554/eLife.46188 PMC6777939

[175] L. Liu , Y. Koo , T. Russell , E. Gay , Y. Li , Y. Yun , PLoS One 2020, 15, e0230335. 32163499 10.1371/journal.pone.0230335 PMC7067464

[176] V. V. Orlova , D. M. Nahon , A. Cochrane , Xu Cao , C. Freund , F. Van Den Hil , C. J. J. Westermann , R. J. Snijder , J. K. Ploos Van Amstel , P. Ten Dijke , F. Lebrin , H.‐J. Mager , C. L. Mummery , Stem Cell Rep. 2022, 17, 1536. 10.1016/j.stemcr.2022.05.022 PMC9287680 35777360

[177] A. Sánchez Alvarado , S. Yamanaka , Cell 2014, 157, 110. 24679530 10.1016/j.cell.2014.02.041 PMC4074550

[178] S. G. Canfield , M. J. Stebbins , B. S. Morales , S. W. Asai , G. D. Vatine , C. N. Svendsen , S. P. Palecek , E. V. Shusta , J. Neurochem. 2017, 140, 874. 27935037 10.1111/jnc.13923 PMC5339046

[179] A. Appelt‐Menzel , A. Cubukova , K. Günther , F. Edenhofer , J. Piontek , G. Krause , T. Stüber , H. Walles , W. Neuhaus , M. Metzger , Stem Cell Rep. 2017, 8, 894. 10.1016/j.stemcr.2017.02.021 PMC5390136 28344002

[180] B. W. Ellis , A. Acun , U. I. Can , P. Zorlutuna , Biomicrofluidics 2017, 11, 024105. 28396709 10.1063/1.4978468 PMC5367145

[181] S. Sances , R. Ho , G. Vatine , D. West , A. Laperle , A. Meyer , M. Godoy , P. S. Kay , B. Mandefro , S. Hatata , C. Hinojosa , N. Wen , D. Sareen , G. A. Hamilton , C. N. Svendsen , Stem Cell Rep. 2018, 10, 1222. 10.1016/j.stemcr.2018.02.012 PMC5998748 29576540

[182] N. Huebsch , B. Charrez , G. Neiman , B. Siemons , S. C. Boggess , S. Wall , V. Charwat , K. H. Jæger , D. Cleres , Å. Telle , F. T. Lee‐Montiel , N. C. Jeffreys , N. Deveshwar , A. G. Edwards , J. Serrano , M. Snuderl , A. Stahl , A. Tveito , E. W. Miller , K. E. Healy , Nat. Biomed. Eng. 2022, 6, 372. 35478228 10.1038/s41551-022-00884-4 PMC10344596

[183] L. Huang , A. Holtzinger , I. Jagan , M. Begora , I. Lohse , N. Ngai , C. Nostro , R. Wang , L. B. Muthuswamy , H. C. Crawford , C. Arrowsmith , S. E. Kalloger , D. J. Renouf , A. A. Connor , S. Cleary , D. F. Schaeffer , M. Roehrl , M.‐S. Tsao , S. Gallinger , G. Keller , S. K. Muthuswamy , Nat. Med. 2015, 21, 1364. 26501191 10.1038/nm.3973 PMC4753163

[184] N. Sone , S. Konishi , K. Igura , K. Tamai , S. Ikeo , Y. Korogi , S. Kanagaki , T. Namba , Y. Yamamoto , Y. Xu , K. Takeuchi , Y. Adachi , T. F. Chen‐Yoshikawa , H. Date , M. Hagiwara , S. Tsukita , T. Hirai , Y.‐S. Torisawa , S. Gotoh , Sci. Transl. Med. 2021, 13, eabb1298. 34233948 10.1126/scitranslmed.abb1298

[185] M. Abulaiti , Y. Yalikun , K. Murata , A. Sato , M M. Sami , Y. Sasaki , Y. Fujiwara , K. Minatoya , Y. Shiba , Yo Tanaka , H. Masumoto , Sci. Rep. 2020, 10, 19201. 33154509 10.1038/s41598-020-76062-w PMC7645446

[186] S. Wiedenmann , M. Breunig , J. Merkle , C. Von Toerne , T. Georgiev , M. Moussus , L. Schulte , T. Seufferlein , M. Sterr , H. Lickert , S. E. Weissinger , P. Möller , S. M. Hauck , M. Hohwieler , A. Kleger , M. Meier , Nat. Biomed. Eng. 2021, 5, 897. 34239116 10.1038/s41551-021-00757-2 PMC7611572

[187] D. C. Hinshaw , L. A. Shevde , Cancer Res. 2019, 79, 4557. 31350295 10.1158/0008-5472.CAN-18-3962 PMC6744958

[188] W. S. Dalton , Drug Resist. Updates 1999, 2, 285. 10.1054/drup.1999.0097 11504502

[189] O. Tredan , C. M. Galmarini , K. Patel , I. F. Tannock , J. Natl. Cancer Inst. Monogr. 2007, 99, 1441. 10.1093/jnci/djm135 17895480

[190] Y. Xiao , D. Yu , Pharmacol. Ther. 2021, 221, 107753. 33259885 10.1016/j.pharmthera.2020.107753 PMC8084948

[191] N. M. Anderson , M. C Simon , Curr. Biol. 2020, 30, R921. 32810447 10.1016/j.cub.2020.06.081 PMC8194051

[192] K. H. Shain , T. H. Landowski , W. S. Dalton , Curr. Opin. Oncol. 2000, 12, 557. 11085455 10.1097/00001622-200011000-00008

[193] V. Salvatore , G. Teti , S. Focaroli , M. C. Mazzotti , A. Mazzotti , M. Falconi , OncoTargets Ther. 2017, 8, 9608. 10.18632/oncotarget.14155 PMC5354757 28030810

[194] S. L. Wood , M. Pernemalm , P. A. Crosbie , A. D. Whetton , Cancer Treat. Rev. 2014, 40, 558. 24176790 10.1016/j.ctrv.2013.10.001

[195] D. Spano , M. Zollo , Clin. Exp. Metastasis 2012, 29, 381. 22322279 10.1007/s10585-012-9457-5

[196] X. Mao , J. Xu , W. Wang , C. Liang , J. Hua , J. Liu , B. Zhang , Q. Meng , X. Yu , S. Shi , Mol. Cancer 2021, 20, 131. 34635121 10.1186/s12943-021-01428-1 PMC8504100

[197] W. Liu , H. Hu , Z. Shao , X. Lv , Z. Zhang , X. Deng , Q. Song , Y. Han , T. Guo , L. Xiong , B. Wang , Y. Zhang , Bone Res. 2023, 11, 4. 36596773 10.1038/s41413-022-00237-6 PMC9810605

[198] Z.‐W. Li , W. S. Dalton , Blood Rev. 2006, 20, 333. 16920238 10.1016/j.blre.2005.08.003

[199] A. Östman , Nat. Med. 2012, 18, 1332. 22961158 10.1038/nm.2938

[200] X. Geng , H. Chen , L. Zhao , J. Hu , W. Yang , G. Li , C. Cheng , Z. Zhao , T. Zhang , Le Li , B. Sun , Front. Cell Dev. Biol. 2021, 9, 655152. 34336821 10.3389/fcell.2021.655152 PMC8319605

[201] E. Sahai , I. Astsaturov , E. Cukierman , D. G. Denardo , M. Egeblad , R. M. Evans , D. Fearon , F. R. Greten , S. R. Hingorani , T. Hunter , R. O. Hynes , R. K. Jain , T. Janowitz , C. Jorgensen , A. C. Kimmelman , M. G. Kolonin , R. G. Maki , R. S Powers , E. Puré , D. C. Ramirez , R. Scherz‐Shouval , M. H. Sherman , S. Stewart , T. D. Tlsty , D. A. Tuveson , F. M. Watt , V. Weaver , A. T. Weeraratna , Z. Werb , Nat. Rev. Cancer 2020, 20, 174. 31980749 10.1038/s41568-019-0238-1 PMC7046529

[202] M. C. Takenaka , G. Gabriely , V. Rothhammer , I. D. Mascanfroni , M. A. Wheeler , C.‐C. Chao , C. Gutiérrez‐Vázquez , J. Kenison , E. C. Tjon , A. Barroso , T. Vandeventer , K. A. De Lima , S. Rothweiler , L. Mayo , S. Ghannam , S. Zandee , L. Healy , D. Sherr , M. F. Farez , A. Prat , J. Antel , D. A. Reardon , H. Zhang , S. C. Robson , G. Getz , H. L. Weiner , F. J. Quintana , Nat. Neurosci. 2019, 22, 729. 30962630 10.1038/s41593-019-0370-y PMC8052632

[203] E. C. Damisah , R. A. Hill , A. Rai , F. Chen , C. V. Rothlin , S. Ghosh , J. Grutzendler , Sci. Adv. 2020, 6, eaba3239. 32637606 10.1126/sciadv.aba3239 PMC7319765

[204] T. Cramer , R. Gill , Z. S. Thirouin , M. Vaas , S. Sampath , F. Martineau , S. B. Noya , P. Panzanelli , T. J. J. Sudharshan , D. Colameo , P. K.‐Y. Chang , P. Y. Wu , R. Shi , P. A. Barker , S. A. Brown , R. C. Paolicelli , J. Klohs , R. A. Mckinney , S. K. Tyagarajan , Sci. Adv. 2022, 8, eabj0112. 35245123 10.1126/sciadv.abj0112 PMC8896802

[205] Y. Shibuya , K. K. Kumar , M. M.‐D. Mader , Y. Yoo , L. A. Ayala , Mu Zhou , M. A. Mohr , G. Neumayer , I. Kumar , R. Yamamoto , P. Marcoux , B. Liou , F. C Bennett , H. Nakauchi , Y. Sun , X. Chen , F. L. Heppner , T. Wyss‐Coray , T. C. Südhof , M. Wernig , Sci. Transl. Med. 2022, 14, eabl9945. 35294256 10.1126/scitranslmed.abl9945 PMC9618306

[206] M. Sochocka , B. S. Diniz , J. Leszek , Mol. Neurobiol. 2017, 54, 8071. 27889895 10.1007/s12035-016-0297-1 PMC5684251

[207] R. M. Ransohoff , D. Schafer , A. Vincent , N. E. Blachère , A. Bar‐Or , Neurotherapeutics 2015, 12, 896. 26306439 10.1007/s13311-015-0385-3 PMC4604183

[208] M. D. Sweeney , S. Ayyadurai , B. V. Zlokovic , Nat. Neurosci. 2016, 19, 771. 27227366 10.1038/nn.4288 PMC5745011

[209] A. Armulik , G. Genové , M. Mäe , M. H. Nisancioglu , E. Wallgard , C. Niaudet , L. He , J. Norlin , P. Lindblom , K. Strittmatter , B. R. Johansson , C. Betsholtz , Nature 2010, 468, 557. 20944627 10.1038/nature09522

[210] A. Ebringer , T. Rashid , Clin. Dev. Immunol. 2006, 13, 41. 16603443 10.1080/17402520600576578 PMC2270745

[211] G. J. Tobón , P. Youinou , A. Saraux , Autoimmun. Rev. 2010, 9, A288. 19944780 10.1016/j.autrev.2009.11.019

[212] M. M. Goldenberg , P T 2012, 37, 175. 22605909 PMC3351877

[213] S. Bandzar , S. Gupta , M. O. Platt , Cell. Immunol. 2013, 286, 45. 24321565 10.1016/j.cellimm.2013.11.003

[214] L. L. Levitsky , M. Misra , Up To Date 2007, 17, 23.

[215] L. Wang , Fu‐S Wang , M. E Gershwin , J. Intern. Med. 2015, 278, 369. 26212387 10.1111/joim.12395

[216] A. H. Cross , R. T. Naismith , J. Intern. Med. 2014, 275, 350. 24444048 10.1111/joim.12203

[217] K. Kakleas , A. Soldatou , F. Karachaliou , K. Karavanaki , Autoimmun. Rev. 2015, 14, 781. 26001590 10.1016/j.autrev.2015.05.002

[218] O. Skog , S. Korsgren , Å. Melhus , O. Korsgren , Diabetes Obes. 2013, 20, 118. 10.1097/MED.0b013e32835edb89 23422243

[219] E. Alard , A.‐B. Butnariu , M. Grillo , C. Kirkham , D. A. Zinovkin , L. Newnham , J. Macciochi , M. Z. I. Pranjol , Cancers 2020, 12, 1826. 32645977 10.3390/cancers12071826 PMC7408985

[220] S. Wang , K. Xie , T. Liu , Front. Immunol. 2021, 12, 690112. 34367148 10.3389/fimmu.2021.690112 PMC8335396

[221] L. Chen , F. Chen , J. Li , Y. Pu , C. Yang , Y. Wang , Y. Lei , Y. Huang , Thorac. Cancer 2022, 13, 889. 35289077 10.1111/1759-7714.14375 PMC8977151

[222] M. Zeltsman , J. Dozier , E. Mcgee , D. Ngai , P. S. Adusumilli , Transl. Res. 2017, 187, 1. 28502785 10.1016/j.trsl.2017.04.004 PMC5581988

[223] L. A. Low , C. Mummery , B. R. Berridge , C. P. Austin , D. A. Tagle , Nat. Rev. Drug Discovery 2021, 20, 345. 32913334 10.1038/s41573-020-0079-3

[224] D. R. Reyes , H. Van Heeren , S. Guha , L. Herbertson , A. P. Tzannis , J. Ducrée , H. Bissig , H. Becker , Lab Chip 2021, 21, 9. 33289737 10.1039/d0lc00963f

[225] X. Cui , C. Ma , V. Vasudevaraja , J. Serrano , J. Tong , Y. Peng , M. Delorenzo , G. Shen , J. Frenster , R.‐T. T. Morales , Elife 2020, 9, e52253. 32909947 10.7554/eLife.52253 PMC7556869

[226] W. Wei , F. Cardes , A. Hierlemann , M. M. Modena , Adv. Sci. 2023, 10, 2205752. 10.1002/advs.202205752 PMC10104638 36782313

[227] S. Palma‐Florez , A. López‐Canosa , F. Moralez‐Zavala , O. Castaño , M. J. Kogan , J. Samitier , A. Lagunas , M. Mir , J. Nanobiotechnol. 2023, 21, 115. 10.1186/s12951-023-01798-2 PMC10053726 36978078

[228] H. M. U. Farooqi , M. A. U. Khalid , K. H. Kim , S. R. Lee , K. H. Choi , J. Micromech. Microeng. 2020, 30, 115013.

[229] B. Schuster , M. Junkin , S. S. Kashaf , I. Romero‐Calvo , K. Kirby , J. Matthews , C. R. Weber , A. Rzhetsky , K. P. White , S. Tay , Nat. Commun. 2020, 11, 5271. 33077832 10.1038/s41467-020-19058-4 PMC7573629

[230] W. Lee , B. Yoon , J. Lee , S. Jung , Y. S. Oh , J. Ko , N. L. Jeon , BioChip J. 2023, 17, 357.

[231] R. Novak , M. Ingram , S. Marquez , D. Das , A. Delahanty , A. Herland , B. M. Maoz , S. S. F. Jeanty , M. R. Somayaji , M. Burt , E. Calamari , A. Chalkiadaki , A. Cho , Y. Choe , D. B. Chou , M. Cronce , S. Dauth , T. Divic , J. Fernandez‐Alcon , T. Ferrante , J. Ferrier , E. A. Fitzgerald , R. Fleming , S. Jalili‐Firoozinezhad , T. Grevesse , J. A. Goss , T. Hamkins‐Indik , O. Henry , C. Hinojosa , T. Huffstater , et al., Nat. Biomed. Eng. 2020, 4, 407.31988458

[232] A. Herland , B. M. Maoz , D. Das , M. R. Somayaji , R. Prantil‐Baun , R. Novak , M. Cronce , T. Huffstater , S. S. F. Jeanty , M. Ingram , A. Chalkiadaki , D. Benson Chou , S. Marquez , A. Delahanty , S. Jalili‐Firoozinezhad , Y. Milton , A. Sontheimer‐Phelps , B. Swenor , O. Levy , K. K. Parker , A. Przekwas , D. E. Ingber , Nat. Biomed. Eng. 2020, 4, 421. 31988459 10.1038/s41551-019-0498-9 PMC8011576

[233] K.‐J. Jang , M. A. Otieno , J. Ronxhi , H.‐K. Lim , L. Ewart , K. R. Kodella , D. B. Petropolis , G. Kulkarni , J. E. Rubins , D. Conegliano , J. Nawroth , D. Simic , W. Lam , M. Singer , E. Barale , B. Singh , M. Sonee , A. J. Streeter , C. Manthey , B. Jones , A. Srivastava , L. C. Andersson , D. Williams , H. Park , R. Barrile , J. Sliz , A. Herland , S. Haney , K. Karalis , D. E. Ingber , et al., Sci. Transl. Med. 2019, 11, eaax5516. 31694927 10.1126/scitranslmed.aax5516

[234] D. E. Ingber , Nat. Rev. Genet. 2022, 23, 467. 35338360 10.1038/s41576-022-00466-9 PMC8951665

[235] D. A. Tagle , Curr. Opin. Pharmacol. 2019, 48, 146. 31622895 10.1016/j.coph.2019.09.007

[236] M. Mastrangeli , S. Millet , C. Mummery , P. Loskill , D. Braeken , W. Eberle , M. Cipriano , L. Fernandez , M. Graef , X. Gidrol , Altern. Anim. Exp. 2019, 36, 481. 10.14573/altex.1905221 31329263

